# OrganoIDNet: a deep learning tool for identification of therapeutic effects in PDAC organoid-PBMC co-cultures from time-resolved imaging data

**DOI:** 10.1007/s13402-024-00958-2

**Published:** 2024-05-28

**Authors:** Nathalia Ferreira, Ajinkya Kulkarni, David Agorku, Teona Midelashvili, Olaf Hardt, Tobias J. Legler, Philipp Ströbel, Lena-Christin Conradi, Frauke Alves, Fernanda Ramos-Gomes, M. Andrea Markus

**Affiliations:** 1 Translational Molecular Imaging, Max-Planck-Institute for Multidisciplinary Sciences, Göttingen, Germany; 2 Miltenyi Biotec B.V. & Co. KG, Bergisch Gladbach, Germany; 3 Department of General, Visceral and Pediatric Surgery, University Medical Center Göttingen, Robert-Koch-Straβe 40, 37075 Göttingen, Germany; 4 Department of Transfusion Medicine, University Medical Center Göttingen, Göttingen, Germany; 5 Institute of Pathology, University Medical Center Göttingen, Göttingen, Germany; 6 Clinic of Hematology and Medical Oncology, Department of Diagnostic and Interventional Radiology, University Medical Center Göttingen, Göttingen, Germany

**Keywords:** PDAC, Organoids, Co-cultures, Gemcitabine, Immunotherapy, Artificial intelligence

## Abstract

**Purpose:**

Pancreatic Ductal Adenocarcinoma (PDAC) remains a challenging disease due to its complex biology and aggressive behavior with an urgent need for efficient therapeutic strategies. To assess therapy response, pre-clinical PDAC organoid-based models in combination with accurate real-time monitoring are required.

**Methods:**

We established stable live-imaging organoid/peripheral blood mononuclear cells (PBMCs) co-cultures and introduced OrganoIDNet, a deep-learning-based algorithm, capable of analyzing bright-field images of murine and human patient-derived PDAC organoids acquired with live-cell imaging. We investigated the response to the chemotherapy gemcitabine in PDAC organoids and the PD-L1 inhibitor Atezolizumab, cultured with or without HLA-matched PBMCs over time. Results obtained with OrganoIDNet were validated with the endpoint proliferation assay CellTiter-Glo.

**Results:**

Live cell imaging in combination with OrganoIDNet accurately detected size-specific drug responses of organoids to gemcitabine over time, showing that large organoids were more prone to cytotoxic effects. This approach also allowed distinguishing between healthy and unhealthy status and measuring eccentricity as organoids’ reaction to therapy. Furthermore, imaging of a new organoids/PBMCs sandwich-based co-culture enabled longitudinal analysis of organoid responses to Atezolizumab, showing an increased potency of PBMCs tumor-killing in an organoid-individual manner when Atezolizumab was added.

**Conclusion:**

Optimized PDAC organoid imaging analyzed by OrganoIDNet represents a platform capable of accurately detecting organoid responses to standard PDAC chemotherapy over time. Moreover, organoid/immune cell co-cultures allow monitoring of organoid responses to immunotherapy, offering dynamic insights into treatment behavior within a co-culture setting with PBMCs. This setup holds promise for real-time assessment of immunotherapeutic effects in individual patient-derived PDAC organoids.

**Supplementary Information:**

The online version contains supplementary material available at 10.1007/s13402-024-00958-2.

## Introduction

Pancreatic Ductal Adenocarcinoma (PDAC), accounting for more than 90% of all pancreatic malignancies, represents one of the deadliest cancer types with increasing incidence [1]. Despite significant advancements in cancer research and treatment options, PDAC continues to exhibit a dismal prognosis with a five-year survival rate below 8% and less than 20% after one year [2, 3]. This can mainly be traced back to its rapid progression, late diagnosis, often already with distant metastasis, as well as to its intrinsic chemo- and radio-resistance. The highly immunosuppressive, nutrient-poor, hypoxic, and desmoplastic tumor microenvironment (TME) contributes to this highly fatal course of disease and to treatment failure as it impairs access to chemical and cellular agents, and promotes chemoresistance and immune escape. In addition, several driver genes such as *KRAS* (found in up to 90% of the cases), *TP53*, *CDKN2A*, or *SMAD4* were identified in PDAC together with many additional mutations, generating an extremely high heterogeneity landscape [4], that not only occurs across patients but can also vary dramatically within individual tumors. This makes it very difficult to find a single therapeutic agent that benefits all PDAC patients. The growing understanding of variances in genetics, treatment response, and aspects of tumor composition in PDAC will help to overcome the challenges of this complex and hard-to-treat disease. Thus, there is an urgent need to not only provide novel therapeutic strategies for PDAC patients but also to establish cell-based preclinical tools that allow the investigation of more efficacious anti-PDAC therapies.

Currently, PDAC therapy includes surgery for resectable tumors, and chemotherapy with the nucleoside analog gemcitabine, plus paclitaxel or FOLFIRINOX, a combination of 5-fluorouracil (5-FU), leucovorin, irinotecan, and oxaliplatin, for patients with advanced PDAC either in neo-adjuvant, adjuvant, or palliative settings, regrettably resulting in only modest improvement in survival [5]. Immune-based therapies have emerged as a promising therapeutic alternative option for various cancer subtypes. In particular, the discovery of immune checkpoint molecules, co-inhibitory receptors expressed on the surface of T cells to negatively regulate T cell-mediated immune responses such as cytotoxic T-lymphocyte-associated protein 4 (CTLA-4), programmed cell death-1 (PD-1) and its ligand programmed death-ligand 1 (PD-L1) have made a huge impact on the clinical use of cancer immunotherapy. Targeting their receptors to stimulate anti-cancer immune responses by applying anti-CTLA-4, anti-PD-1 and anti-PD-L1 monoclonal antibodies and other immune checkpoint inhibitors has been markedly successful in treating numerous malignancies such as melanoma, non-small-cell lung cancer, and ovarian cancer [6]. However, in PDAC its efficacy is limited since the pancreatic TME is considered extremely immunosuppressive and generally lacks immune infiltration [7]. So far targeting PD-L1, overexpressed in several tumors, has shown benefits in different tumors [8, 9]. Most recently, the combinatorial treatment of PDAC using the monoclonal anti-PD-L1 antibody Atezolizumab together with other therapies such as gemcitabine or neoantigen-based vaccines, has been successful in several studies and clinical trials [10–12]. Thus, this denotes the potency that this immune checkpoint PD-L1 inhibitor exerts as an adjuvant PDAC treatment.

However, despite these recent advances, not all patients respond to current immunotherapies. Treatment failure may be explained at many different levels that include a low number of immunogenic antigens, malfunctioned antigen presentation, and/or the expression of alternative immune checkpoint molecules [13]. Tools to allow the unbiased and systematic analysis of T cell-mediated tumor recognition on an individual patient basis would greatly contribute to predicting whether an individual patient will be sensitive to immunotherapy. Here, patient-derived organoids (PDOs) in 3D cell culture which can be generated from human primary tumor material, for instance primary tumors from surgical interventions, fine needle biopsies from non-resectable PDAC patients, and from metastases appear as an attractive model allowing drug testing on a large scale. PDOs have already demonstrated the ability to predict clinical response to chemotherapy ex vivo, thus serving as a pivotal stepping stone toward personalized cancer treatments [14–18]. When compared to traditional 2D cell cultures, PDOs faithfully recapitulate the genetic landscape, the marked heterogeneity described in patients, and the biological intricacies of solid tumors, including PDAC, in an in vitro approach [19–22].

While purely epithelial organoid cultures hold great promise for assessing chemotherapeutic agents, evaluating the efficacy of immunotherapies faces challenges including the lack of immune cells that are important to assess an efficient immune response [23]. To address the research need, the establishment of PDO heterocellular cultures with the immune system has become a hot topic of research [24]. Specifically for PDAC organoids, some studies have already succeeded in immune cells/PDO co-cultures. Holokai et al. [25] have shown the co-culture of murine and human PDAC organoids with autologous myeloid-derived suppressor cells (MDSCs) and cytotoxic T lymphocytes (CTLs) by mixing immune and organoid populations in matrigel. In addition, Knoblauch et al. [26] presented an optimized method from Dijkstra et al. [27], enabling the direct contact of PDOs with HLA-matched PBMCs for 48 h. Moreover, there is a need to optimize co-culture protocols to enable precise image-data collection with minimal motility of the organoids and stable Z-positions in the co-culture. So far, most of the drug testing studies only assess organoid viability at the endpoint, such as the luminescence ATP-based CellTiter-Glo® cell viability assay, as well as ATP-independent assays like Cytotox-Glo™ cytotoxicity and CyQUANT™ assays, which provide reproducible results [28].

To assess the real-time effect of immunotherapies in PDAC organoids/PBMCs co-cultures, we developed OrganoIDNet, an innovative algorithm that uses the power of artificial intelligence (AI) to analyze live cell imaging data to assess organoid growth. This allows accurate monitoring of the longitudinal response of PDAC organoids to chemotherapy in organoid cultures and immunotherapy in a novel organoid/PBMC co-culture set-up. For assessment of the PD-L1 inhibitor Atezolizumab, we established an optimized sandwich protocol that enabled a stable Z-position acquisition and thus an enhanced image quality. This platform addresses the complexities of organoids, since OrganoIDNet not only identifies organoids within images of the culture over time but also depicts size-dependent effects of drugs on organoids, allows eccentricity measures and distinguishes between healthy and unhealthy status. This presents a significant step forward in standardizing in vitro immunotherapy testing methodologies for PDAC. Ultimately, this approach will support the pre-clinical testing of personalized immunotherapy-based treatment strategies for PDAC patients, marking a significant leap forward in cancer therapy.

## Materials and methods

### Patient PDAC tissue

All research involving human PDAC material obtained from patients participating in the Molecular Pancreas Program (MolPAC) at the University Medical Center of Göttingen, which includes the generation and utilization of PDAC PDOs, has received approval from local regulatory authorities (approval references: 11/5/17, 22/8/21Ü, and 2/4/19).

### Murine tumor tissue

For mouse-derived PDAC tissue, 5 × 10^5 KPC cells were orthotopically implanted into the head of the pancreas of C57BL/6 J mice as described before [29]. Tumors developed within 2–3 weeks post-implantation and were excised during the section for organoid formation. KPC cells were cultured in Dulbecco’s Modified Eagle’s Medium (DMEM, Gibco) with 10% Fetal Calf Serum (FCS, Gibco).

KPC cells were established from tumors of the KPC mouse that contains a conditional point mutation in the transformation-related protein *TP53* gene (*TP53R172H*), and a point mutation in the *KRAS* gene (*KRASG12D*) both of which generate non-functional proteins [30]. The cells were kindly provided by Prof. Dr. Volker Ellenrieder (Klinik für Gastroenterologie, Gastrointestinale Onkologie und Endokrinologie, University Medical Center, Göttingen, Germany).

All animal procedures were performed in compliance with the guidelines of the ARRIVE, European Directive (2010/63/EU), and the German ethical laws and were approved by the administration of Lower Saxony, Germany (approval number G20.3527).

### Medium preparation

#### Digestion medium

Per 100 ml: 12 mg Collagenase type I (Sigma), 12 mg Dispase II (Sigma), and 1 ml of 10% FCS (Gibco) were added to 99 ml of DMEM (Gibco).

#### Human organoid growth medium (HOGM)

Per 50 ml of HOGM: 25 µl A83-01 (1 mM, Tocris), 50 µl Human Epidermal Growth Factor (hEGF; 500 µg/ml, Invitrogen), 50 µl human Fibroblast Growth Factor-10 (hFGF-10; 100 mg/ml, Peprotech), 50 µl Gastrin I (100 µM, Sigma), 125 µl N-acetylcysteine (500 mM, Sigma), 500 µl Nicotinamide (1 M, Sigma), 1 ml B-27 supplement (50x, Gibco), 100 µl Primocin (50 mg/ml, InvivoGen), 25 ml of Wnt3a-, 5 ml R-spondin and 50 µl of Noggin-conditioned media were diluted in 19 ml of organoid splitting medium (1x Glutamax (Gibco), 1x HEPES (Gibco), 1 ml 1x Primocin (InvivoGen), 30% Bovine Serum Albumin (BSA) (Sigma) (diluted in Advanced DMEM/F12 medium (AdDMEM/F12, Gibco). For the initial seeding, splitting, or thawing, 10 µM Y-27632 Rho Kinase Inhibitor (Sigma) was added to the organoid medium.

#### Murine organoid growth medium (MOGM)

Per 50 ml of MOGM: 5 µl murine Epidermal Growth Factor (mEGF; 500 µg/ml, Invitrogen), 50 µl murine Fibroblast Growth Factor-10 (mFGF-10; 100 µg/ml, Peprotech), 5 µl Gastrin I (100 µM, Sigma), 125 µl N-acetylcysteine (500 mM, Sigma), 500 µl Nicotinamide (1 M, Sigma), 1 ml B-27 supplement (50x, Gibco), 5 ml R-spondin and 5 ml of Noggin-conditioned media were diluted in 38.3 ml of organoid splitting medium (1x Glutamax (Gibco), 1x HEPES (Gibco), 1% P/S diluted in AdDMEM/F12 (Gibco). For the initial seeding, splitting, or thawing, 10 µM Y-27632 Rho Kinase Inhibitor (Sigma) was added to the organoid medium.

#### Wnt3a-, R-Spondin- and Noggin-conditioned media

For the preparation of Wnt3a-conditioned media, the L-Wnt3A cell line (ATCC® CRL-2647™) was acquired from ATCC and cultured in adherence to the guidelines provided by the manufacturer. A detailed protocol can be found in the work of Wilson et al. [31]. To generate R-spondin- and Noggin-conditioned media, 293T-HA-Rspol-Fc (obtained from Calvin Kuo’s group at Stanford University) and HEK293-mNoggin-Fc (sourced from AG Florian Greten and AG Herner Farin at CSH Frankfurt) cell lines were utilized. The preparation followed the protocol outlined by Klemke et al. [32]. Specifically, 293T-HA-Rspol-Fc or HEK293-mNoggin-Fc cells were thawed and seeded in 175 cm^2^ flasks with growth medium (comprising 500 ml DMEM (Gibco), 60 ml FCS, and 5 ml P/S) supplemented with 300 µg/ml Zeocin (Invitrogen) or 500 µg/ml Geneticin (Gibco), respectively. Upon reaching confluency, cells were expanded into eight (for 293T-HA-Rspol-Fc cells) or six (for HEK293-mNoggin-Fc cells) 175 cm^2^ flasks using antibiotic-free medium. To preserve the cells, one flask from each condition was aliquoted and supplemented with Zeocin or Geneticin before freezing. Subsequently, upon reaching confluency, the antibiotic-free medium was replaced with 50 ml of conditioning medium (AdDMEM/F12 medium supplemented with 1x Glutamax, 1 M HEPES (Invitrogen), and 1% P/S). After one week of culturing, the conditioned medium was collected, centrifuged (500 g, 4 °C, 5 min), pooled from all the flask supernatants, and then filter-sterilized using a 0.22 μm filter. The resulting R-spondin- or Noggin-conditioned media were collected into 15 ml conical tubes (5 ml per tube) and stored at -20 °C.

#### Organoid passaging medium (OPM)

Per 500 ml: 5 ml 100x Glutamax (Gibco), 5 ml 1 M HEPES (Gibco) and 1% P/S were added to 500 ml AdDMEM/F12 (Gibco).

#### PBMCs culture medium

RPMI medium (Gibco) supplemented with 10% FCS, 50 µM β-mercaptoethanol (Gibco), and 1% P/S.

### PDAC organoid establishment and culturing

Two human organoid cultures were established from primary tumors obtained from two independent PDAC patients (described on Sect. 2.1) during surgical intervention, designated Human Organoid 1 (HO1) and Human Organoid 2 (HO2). Human PDAC organoids (PDAC PDOs) were generated from PDAC specimens following standard organoid cell culture methods established as described before [33]. PDAC tissue was extracted from the tumor bulk following resection and confirmation of a PDAC diagnosis. Additionally, Murine Organoid 1 (MO1) and Murine Organoid 2 (MO2) were derived from tumor tissue obtained from two different KPC-tumor-bearing C57BL/6J mice (described in Sect. 2.2). Generation and culturing of both murine and human organoids followed the Tuveson Laboratory Murine and Human Organoids Protocols, accessible at http://tuvesonlab.labsites.cshl.edu/wpcontent/uploads/sites/49/2018/06/20170523_OrganoidProtocols.pdf. In brief, freshly excised tumor tissue was cut into small fragments and resuspended in the digestion medium for enzymatic digestion. After obtaining a cell suspension, a 50 µl mixture of suspended cells with Growth Factor Reduced (GFR) Matrigel (Corning) was added to a preheated 24-well plate. Following the solidification of the dome through a 15-min incubation, the respective organoid growth medium for MOGM or HOGM was applied to the top of the dome. Human and mouse organoid formation was microscopically observed after 3 days of culturing at 37 °C under a humidified atmosphere of 5% CO_2_. The organoids were utilized for experimentation after at least 3 passages.

### Chemotherapy treatment of organoids

On day zero, fully matured organoids were harvested and subjected to mechanical dissociation using a 200 µl tip affixed to a 10 ml serological pipette. A single-cell suspension was subsequently obtained by incubating the organoids for 3 min with Trypsin/EDTA (0.25%/0.02% (w/v) in PBS) solution at 37 °C. Following this, a cell density of 1 × 10^4 cells was mixed with 15 µl of Matrigel and plated in 48-well plates using the murine (MOGM) or human organoid medium (HOGM), as outlined in the protocol available at http://tuvesonlab.labsites.cshl.edu/wpcontent/uploads/sites/49/2018/06/20170523_OrganoidProtocols.pdf, with Y-27632 supplementation.

After 3 days, serial dilutions of gemcitabine (Gemcitabine Hexal^(R)^, 40 mg/ml; ranging from 3 nM to 100 nM) were prepared in murine or human organoid medium and added to the matured organoids. Live imaging to observe the effects of gemcitabine on PDAC organoids was performed for 4 days, after which endpoint ATP levels were measured using the CellTiter-Glo^(R)^ 2.0 reagent (Promega).

### Human and murine PBMC isolation and pre-activation

Blood was collected from healthy C57BL/6J mice via cardiac puncture following euthanasia. Human cells were isolated from Leukocyte Reduction System (LRS) chambers of the local blood bank (UMG ethics approval 29/07/23). After LRS processing, PBMCs of healthy human thrombocytes from HLA-A*02:01 donors and of healthy mice were isolated by density gradient centrifugation utilizing SepmateTM tubes (Stemcell Technologies, 85450 for human and Stemcell Technologies, 85415 for mouse samples) containing Lymphocyte Separation Media (1,077 g/ml, Anprotec), following the manufacturer’s instructions.

The resulting PBMCs were counted and seeded at a density of 1 × 10^5 cells per well in a 96-well round bottom plate in PBMCs medium (RPMI medium (Gibco), supplemented with 10% FCS, 50 µM β-mercaptoethanol (Gibco), and 1% P/S). Following an overnight incubation, murine PBMCs were pre-activated by suspension in LymphoGrow II medium (Cytogen), while human PBMCs were pre-activated by adding 10 µl of ImmunoCult™ Human CD3/CD28 T cell Activator (StemCellTechnologies) per 1 ml of PBMCs medium. After a 24 h pre-activation period, both human and murine PBMCs were used for human and mouse organoid co-cultures, respectively.

### Human- and mouse-derived pancreatic cancer/immune cell co-cultures

Murine and human organoids were prepared as described in Sect. 2.4. On day 3, matured organoids were carefully collected using a 200 µl tip (tip end cut) attached to a 10 ml serological pipette and resuspended in organoid passaging medium to remove the Matrigel. After 3-min centrifugation at 300 g, the medium was removed, and 20 µl of the passaging medium containing pre-activated PBMCs (2.6) was added.

To prepare the plate for co-culture, 30 µl of 25% Matrigel (diluted in DMEM 1x) was added to each well of a 96-well plate. After solidification of the Matrigel through a 15-minute incubation at 37 °C, 20 µl of the PBMCs-organoids mixture was added on top of the first Matrigel layer. Subsequently, an equivalent concentration of Matrigel was added on top of the mixture. Following this, 200 µl of a 50% mixture of MOGM or HOGM and 50% PBMCs culture medium was added on top of the solidified Matrigel layer. For anti-PD-LI treatment, 0.1 mg/ml Atezolizumab (Atezolizumab Tecentriq^(R)^ 1200 mg/20 ml) was added to the 50% MOGM or HOGM/ 50% PBMCs medium.

### Live cell imaging

The growth and viability of organoids and organoid co-cultures were monitored with the live-cell imaging system Incucyte^R^ S3 (Sartorius, Germany). Phase-contrast images were acquired every 4 h up to 100 h using the Organoid mode.

### OrganoIDNet analysis

Following image acquisition with the Incucyte imaging system, bright field (BF) images were analyzed using an in-house developed deep-learning-based analysis software called OragnoIDNet. Briefly, we trained a StarDist model [34] on a custom dataset consisting of manually annotated BF images from the Incucyte imaging system. This dataset featured both human and mouse PDAC organoids, with and without PBMCs. This model was then employed to segment the organoids. Post-segmentation, organoids were categorized based on a mean pixel intensity threshold as either healthy or unhealthy. Additionally, for each condition, the combined areas of all segmented organoids were computed and then size-categorized into five bins: Tiny, Small, Medium, Large, and Huge. Moreover, the deviation of the organoid shape from a perfect circle was measured as Eccentricity, with a value of zero denoting a perfect circle. The analysis output from OrganoIDNet was further correlated with the current organoid analysis standard assay, CellTiter-Glo assay (for drug screening). The OrganoIDNet image analysis software we developed is available to researchers upon reasonable request and discussion with the corresponding author.

### CellTiter-Glo analysis

Cell viability was assessed utilizing the CellTiter-Glo assay (Promega). Following stimulation with either chemotherapeutic agents or Atezolizumab monitored by live cell imaging, 80 µl of CellTiter-Glo reagent was added to each well of a 96-well plate. Subsequently, the plate was mixed for 2 min, followed by a 10 min incubation at room temperature (RT) in the dark to stabilize the luminescence signal. The acquired luminescence signal was then recorded using the multimode plate reader CLARIOstar (BMG LABTECH, Germany) and analyzed by MikroWin 2000 lite Version 4.43.

### Immunofluorescence staining of organoids

Murine PDAC organoids were stained according to the protocol by Yan et al. [35]. First, PDAC organoids were gently separated from the Matrigel matrix using cold PBS/0.1% BSA (PBSB). The absence of Matrigel was confirmed by visual inspection after incubating the suspension in PBS on ice. The organoids were then fixed in 4% paraformaldehyde (PFA) for 30 min. Fixative was removed by rinsing with PBS, and the whole-fixed organoids were stored in PBS at 4 °C until immunofluorescence staining.

Organoids were blocked using PBSDT (PBS supplemented with 0.5% Triton X-100) to minimize nonspecific binding. The following primary rabbit antibodies were used: a polyclonal anti-SRY-Box Transcription Factor 9 (SOX9; Sigma,1:600, ab5535) and a monoclonal anti-Cytokeratin 19 (CK19; Abcam,1:200). The antibodies were diluted in fresh PBSDT solution and applied to the organoids for incubation at 4 °C for 48 h. Organoids were subsequently washed with PBSB (0.1% BSA in PBS) and secondary anti-rabbit antibody Alexa Fluor 546 (Invitrogen, 1:200) was added and incubated for 2 h. Following further 2 washing steps, Hoechst 33342 fluorescent dye (Invitrogen, 1:5000) was added for 15 min at RT for nuclear staining. Finally, the organoids were mounted on glass slides using Acqua-Poly/Mount (Polysciences) mounting media. Imaging was performed using a Zeiss LSM880 confocal laser scanning microscope (CLSM, Carl Zeiss Microscopy GmbH) with a 40x oil objective lens. Alexa Fluor 546 was excited at 561 nm, and Hoechst dye was excited at 405 nm. Single plane and 3D images from microscopy Z-stacks were processed and analyzed using IMARIS 9.1.2 software.

### Flow cytometry analysis of PDAC organoids before and after co-cultures

For flow cytometric phenotyping, the cells were resuspended in 1x PBS/0.5% BSA/2 mM EDTA (PEB) buffer. Up to 10^6 cells were resuspended in 100 µl per staining, transferred to a 96-well plate, and centrifuged at 300 g for 5 min. The pellets were resuspended in 35 µl PEB buffer and 5 µl human FcR Blocking Reagent (Miltenyi Biotec,130-059-901) was added to each well. The sample was mixed, followed by the addition of the different fluorescent labeled primary antibodies for staining (1 µl each; see Table 1), mixed again and incubated for 10 min at 4 °C. Unbound antibodies were removed by washing twice with 250 µl of PEB. The pellets were then resuspended in a 150 µl PEB buffer. For identification of dead cells, propidium iodide (1 µg/ml) was added immediately before sample acquisition. Data was acquired on a MACSQuant Analyzer 16 and data analysis was performed with MACSQuantifyTM software (both Miltenyi Biotec). Gating strategy for PD-L1 expression from immune cell infiltration into human organoids in co-cultures is shown in supplementary Fig. S3, and from non-immune cells in supplementary Fig. S4.

**Table 1:** List of antibodies (Miltenyi Biotec) used for flow cytometric analysis

Target	Conjugate	Catalog number
EpCAM	VioGreen	130-111-005
CD45	PerCP-Vio700	130-110-636
PD-L1	PE	130-122-809
CD3	VioGreen	130-113-142
CD8	APC-Vio770	130-110-681
CD56	VioBright B515	130-114-552

### Statistical analysis

Statistical analysis was performed with GraphPad Prism 9. All presented data are expressed as means with corresponding standard errors of the means (SEMs). Biological samples were mean ± SEM of technical replicates. Comprehensive information regarding the significance tests, including the number of replicates, and associated p values can be found in the respective figure legends.

## Results

### Establishment of PDAC organoids from human and mouse primary tumor tissue

**Fig. 1:**
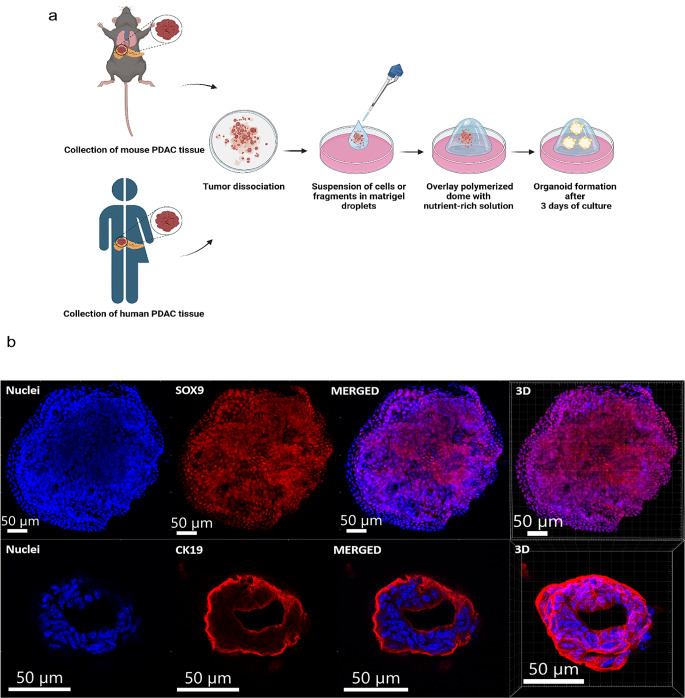
Workflow of the establishment of organoids and validation of KPC-derived mouse PDAC organoids (**a**) Schematic overview of the generation of murine and human PDAC organoids (created with BioRender.com). (**b**) Immunofluorescence images of KPC-tumor derived mouse organoids (MO) stained for SOX9 (upper panel, red) and CK19 (lower panel, red) expression. Nuclei are shown by Hoechst staining in blue. Scale bars: 50 µM

Primary tumor tissue samples were obtained from two KPC-murine tumors or human tumor tissue collected from PDAC patients during surgical resection. The procedure for dissociation and organoid preparation was identical for both types of organoids. After mechanical dissociation and enzymatic digestion of tumor tissue, PDAC organoids were prepared by mixing the resulting small tumor fragments with Matrigel and depositing them as domes (Fig. 1a). After 3 days of culture in their respective organoid growth medium, human or mouse organoids were formed exhibiting different morphologies ranging from spheroid-like to organoids containing a network of lumenal structures, as described before [36]. Similar to the description by Low et al. [37], in mouse organoids we observed a solid organoid subtype, consisting of multiple cellular layers and no presence of a lumen (Fig. 1b, upper panel), and a glandular phenotype, characterized by a single layer of epithelial cells with the lumen in the center (Fig. 1b, lower panel).

Immunofluorescence staining of the KPC-murine tumor-derived organoids confirmed the formation of PDAC organoids, as shown by the expression of two biomarkers, SRY-Box Transcription Factor 9 (SOX9) [38], a lineage marker for pancreatic formation (Fig. 1b, upper panel), and cytokeratin 19 (CK19) [39, 40], a biomarker for epithelial cancer cells (Fig. 1b, lower panel). In the case of patient-derived organoids (PDOs), both organoids, HO1 and HO2, used in subsequent experiments were genetically analyzed by using an RNA sequencing (RNA-seq) protocol revealing mutations in *KRAS, TP53, and SMAD4* (Table 2), consistent with the mutations described in PDAC primary tumors [41].

**Table 2:** Overview of the mutational profile of human PDAC organoids used in this study

Organoid Name	Gene	Gene Status	Gene Variant	% Gene Variant
Human Organoid 1	KRAS	pathogen	p.Gly12Val	44%
	TP53	pathogen	p.Cys176Trp	99%
	CDKN2A	WT	WT	NA
	ARID1A	WT	WT	NA
	SMAD4	pathogen	p.Arg361His	99%
	BRCA1	WT	WT	NA
	BRCA2	WT	WT	NA
Human Organoid 2	KRAS	pathogen	p.Gly12Asp	96%
	TP53	pathogen	p.Cys242Gly	98%
	CDKN2A	pathogen	p.Arg58Ter	97%
	ARID1A	WT	WT	NA
	SMAD4	VUS	p.Arg531Trp	98%
	BRCA1	WT	WT	NA
	BRCA2	WT	WT	NA

WT: Wild Type; NA: Not Applicable; VUS: Variant of uncertain significance

### Assessment of PDAC organoid growth kinetic in response to chemotherapy using OrganoidIDNet

**Fig. 2:**
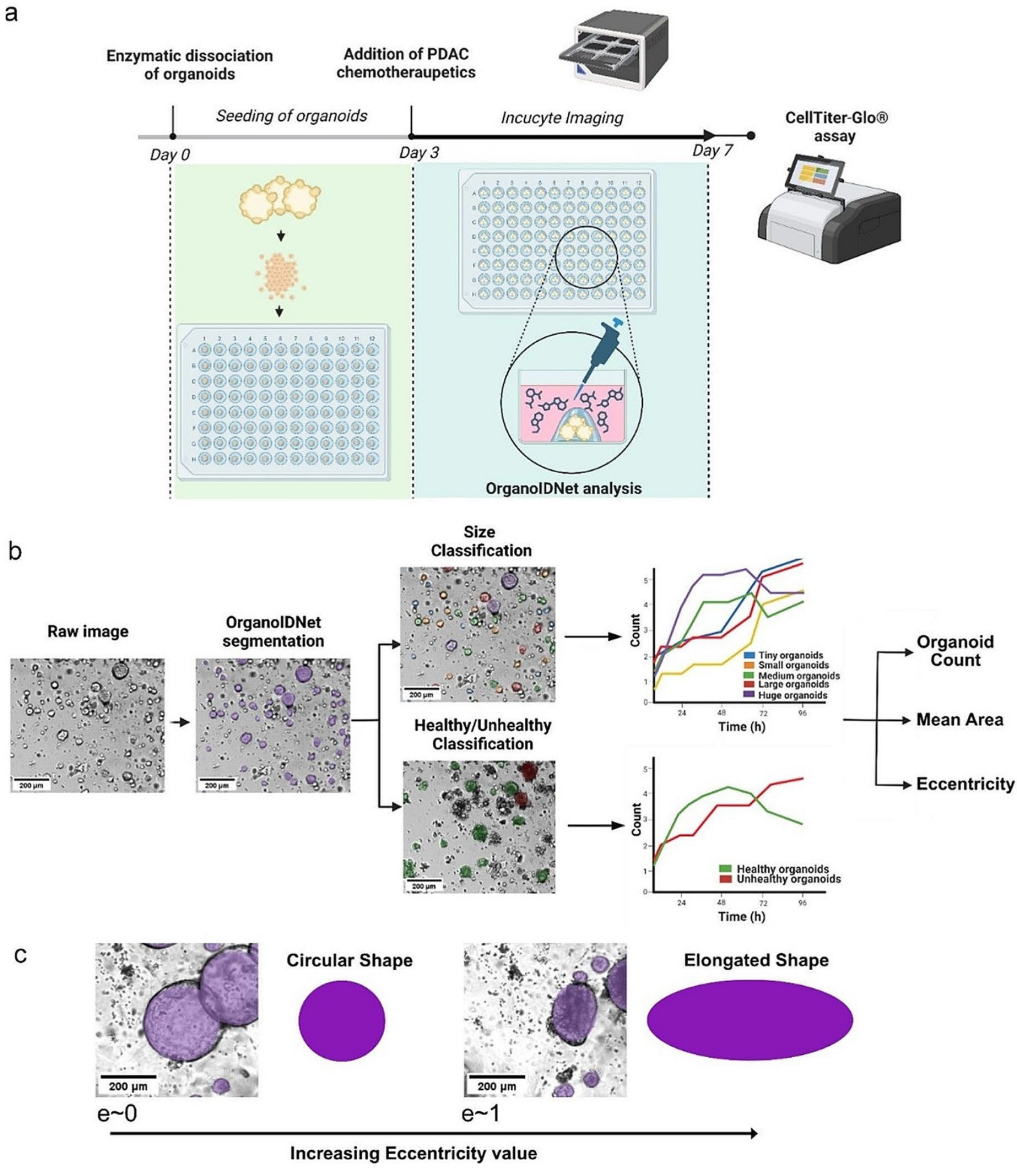
Organoid analysis platform for assessing real-time PDAC response to chemotherapeutics (**a**) Establishment of a protocol for assessing PDAC organoid response to treatment with gemcitabine using OrganoIDNet on live cell imaging data and cell viability endpoint assay by CellTilter-Glo©. (**b**) OrganoIDNet analysis includes the segmentation of individual organoids from raw images and subsequent classification. This differentiates the organoids based on both size categories (from Tiny to Huge organoids) and pixel intensity, which allows distinction between healthy and unhealthy organoids. Organoids that exhibit a mean intensity value below the threshold of 50 are categorized as unhealthy. Counts of different size organoids and healthy/unhealthy ratio were thus assessed over time. (**c**) Eccentricity measures of organoids’ reaction to therapy with values between zero (defining a perfect circular shape) and one (defining an elongated shape). All the plots were normalized to the initial conditions. Scale bar: 200 µM. Images were created using BioRender.com

As organoids are highly heterogeneous, we clustered the organoids based on common-size metrics, to assess how different-sized organoids react to chemotherapy. For this purpose, 3 days after organoid formation, ranging from Tiny to Huge sizes, we incubated the cultures 4 days with different concentrations of gemcitabine, and acquired images of the organoids over this time by live cell imaging using the Incucyte system, which were subsequently analyzed by the OrganoIDNet algorithm. To ascertain the reliability of this approach, we conducted a CellTiter-Glo® assay as an endpoint experiment (Fig. 2a) to confirm the changes in viability obtained by OrganoIDNet (Fig. 2b).

As an initial step for the development of OrganoIDNet, we trained a StarDist model [32] using our custom dataset that included manually annotated images obtained from the Incucyte® live-imaging system. This dataset encompassed images from both human and mouse PDAC organoid models with and without PBMCs. Subsequently, we applied this model to identify and monitor organoids over time from images of treated PDAC organoids. Previous studies have associated the darkness of organoids in bright-field imaging with an unhealthy state [42, 43]. Following segmentation, the organoids were therefore categorized based on pixel intensity, classifying them as either healthy or unhealthy (supplementary VIDEO S1). In this way it was for instance possible to detect merging organoid events, demonstrating how OrganoIDNet recognizes such events as expansion of area and reduction of counts (supplementary VIDEO S2). Moreover, for each well, we calculated the combined areas of all segmented organoids and categorized them into five size bins: Tiny, Small, Medium, Large, and Huge (Fig. 2b and supplementary VIDEO S3). Organoids belonging to the category “Tiny” have a threshold set at the 20th percentile of the combined organoid areas, while those in the “Small,” “Medium,” “Large” and “Huge” categories have thresholds set at the 40th, 60th, and 80th percentiles, respectively, providing distinct size categories. This approach allowed a kinetic evaluation of the occurrence of organoids at an unhealthy state up to death, as well as of size-dependent responses to treatment. Based on the deviation of a curve or orbit from circularity, termed eccentricity, we also evaluated the organoids’ altered shape in response to treatment (Fig. 2c).

**Fig. 3:**
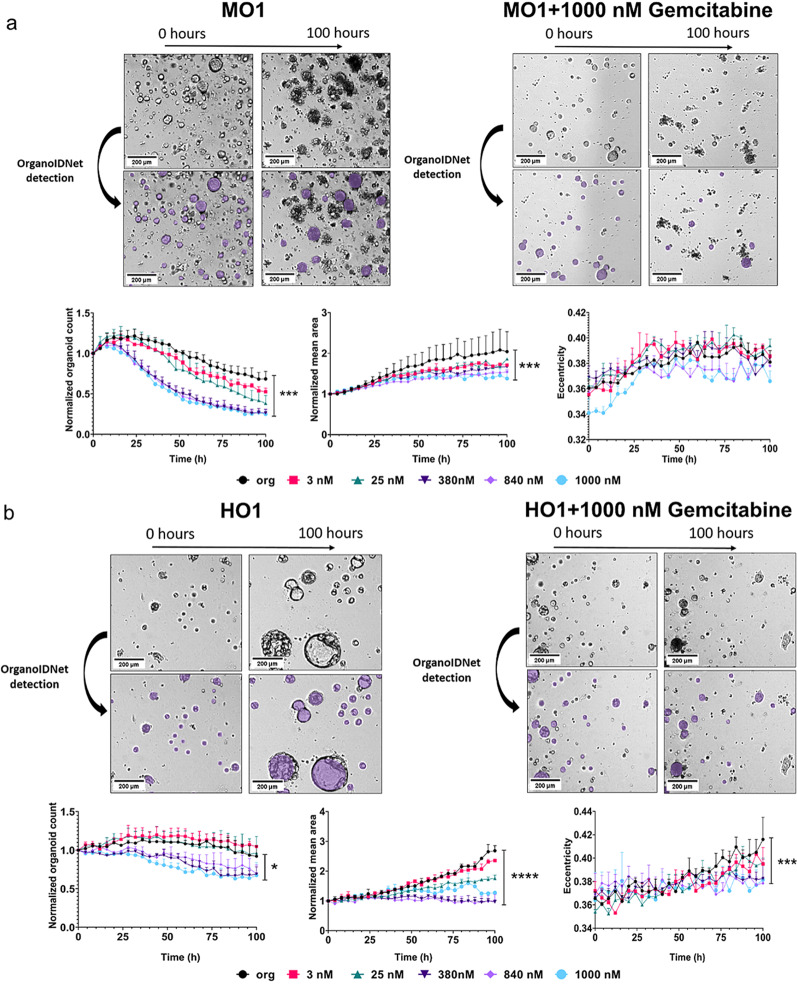
OrganoIDNet analysis reveals gemcitabine-induced toxicity in PDAC organoids. (**a**) Comparison between untreated (left) and treated mouse PDAC organoids (right). Upper panel: representative images of MO1; Lower panel: OrganoIDNet quantification of the number, average area, and eccentricity of organoids over time in response to different concentrations of gemcitabine. (**b**) Comparison between untreated (left) and treated human organoids (right). Upper panel: representative images of HO1; Following 100 h of incubation with 1000 nM gemcitabine both murine and human organoid cultures show less detectable organoids and a substantial amount of cell debris (right). Lower panel: OrganoIDNet quantification of the number, average area, and eccentricity of organoids over time in response to different concentrations of gemcitabine. All parameters were normalized to initial time and data are presented as mean ± SEM of two biological samples, with two technical replicates each. * *p* < 0.05, ** *p* < 0.01, *** *p* < 0.001, **** *p* < 0.0001 (Two-way ANOVA followed by Dunnett’s comparisons). Scale bars: 200 µM

To assess the impact of chemotherapy on PDAC organoids, we analyzed the effect of different concentrations of gemcitabine, ranging from 3 nM to 1000 nM, on proliferation, morphology and health status of organoids. By applying OrganoIDNet to the collected imaging data we were able to assess organoid growth over time. We observed a different response of mouse and human organoids to gemcitabine. In mouse organoids we observed a chemotherapy concentration dependent reduction in organoid count and mean area. Higher concentrations of gemcitabine (380 nM and above) showed a drastic reduction in organoid count as early as 24 h of incubation in comparison to untreated or lower gemcitabine concentrations. 100 h after treatment this difference reached significance in both parameters, organoid count and area, with a growth reduction of about 75% and area decrease of about 80% when compared to untreated organoids (Fig. 3a). We could not detect any changes in eccentricity upon gemcitabine treatment. Regarding the human model, we observed a different trend, where 380 nM gemcitabine elicited a decrease in organoid count and mean area only after 52 h of incubation. A decrease of 30% in organoid counts and ~ 60 to 65% decrease in organoid mean area were observed with the two highest gemcitabine concentrations (840 nM and 1000 nM) at the 100 h mark, as compared to untreated organoids (Fig. 3b). While untreated organoids and those treated with lower gemcitabine concentrations showed an increase in eccentricity, organoids treated with high concentrations of chemotherapy (840 nM and 1000 nM) did not change their morphology over time (Fig. 3b).

**Fig. 4:**
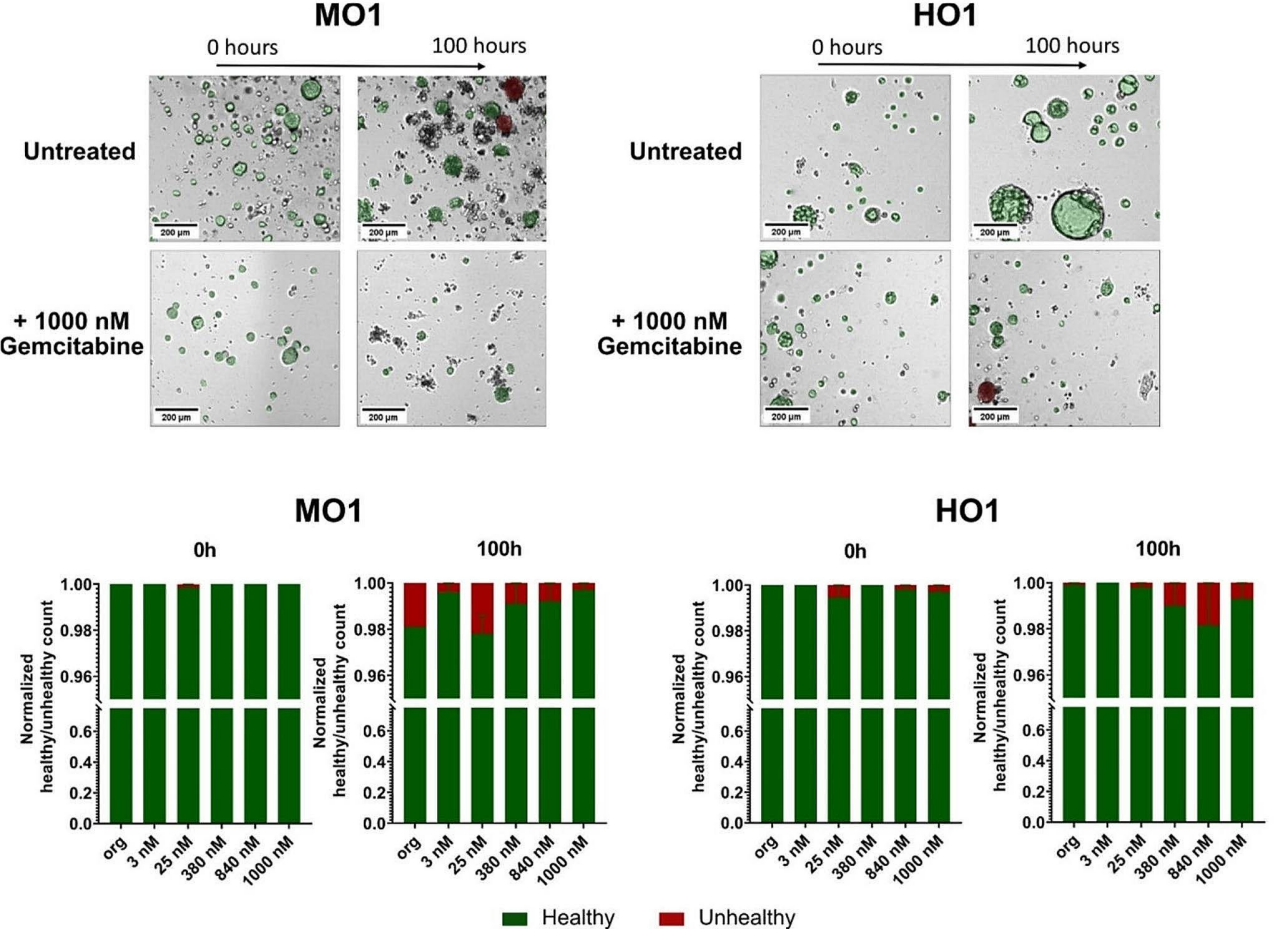
OrganoIDNet analysis shows no effect of gemcitabine on murine and human organoid health status. Representative images (upper panel) of healthy (green) and unhealthy (red) organoids in the mouse (left) and human (right) model in response to 1000 nM gemcitabine (lower panel) in comparison to untreated organoids, using the darkness parameter, which indicates the health status of the organoid. Following 100 h of incubation with 1000 nM gemcitabine both murine and human organoid cultures show less detectable organoids and a substantial amount of cell debris. Lower panel: Quantification of healthy (green) and unhealthy (red) organoids is shown in response to different concentrations of gemcitabine. All parameters were normalized to counts at initial time and data are presented as mean ± SEM of two biological samples, with two technical replicates each. Scale bars: 200 µM

A further important aspect of our analysis was to evaluate the healthy status of the organoids based on the brightness values observed in bright-field microscopy. We used pixel intensity analysis to classify organoids into two groups: dark organoids, indicating unhealthy cells, and bright organoids, representing healthy organoids. We found a slight increased number of unhealthy organoids over time (~ 1–2%), however there was no significant difference between untreated and treated organoids in both murine and human models (Fig. 4, lower panel). This can be explained by the fact that gemcitabine leads to an almost complete killing of organoids which results in debris rather than “unhealthy” (dark) organoids (Fig. 4a, b, upper panels), which cannot be detected by the OrganoIDNet algorithm.

**Fig. 5:**
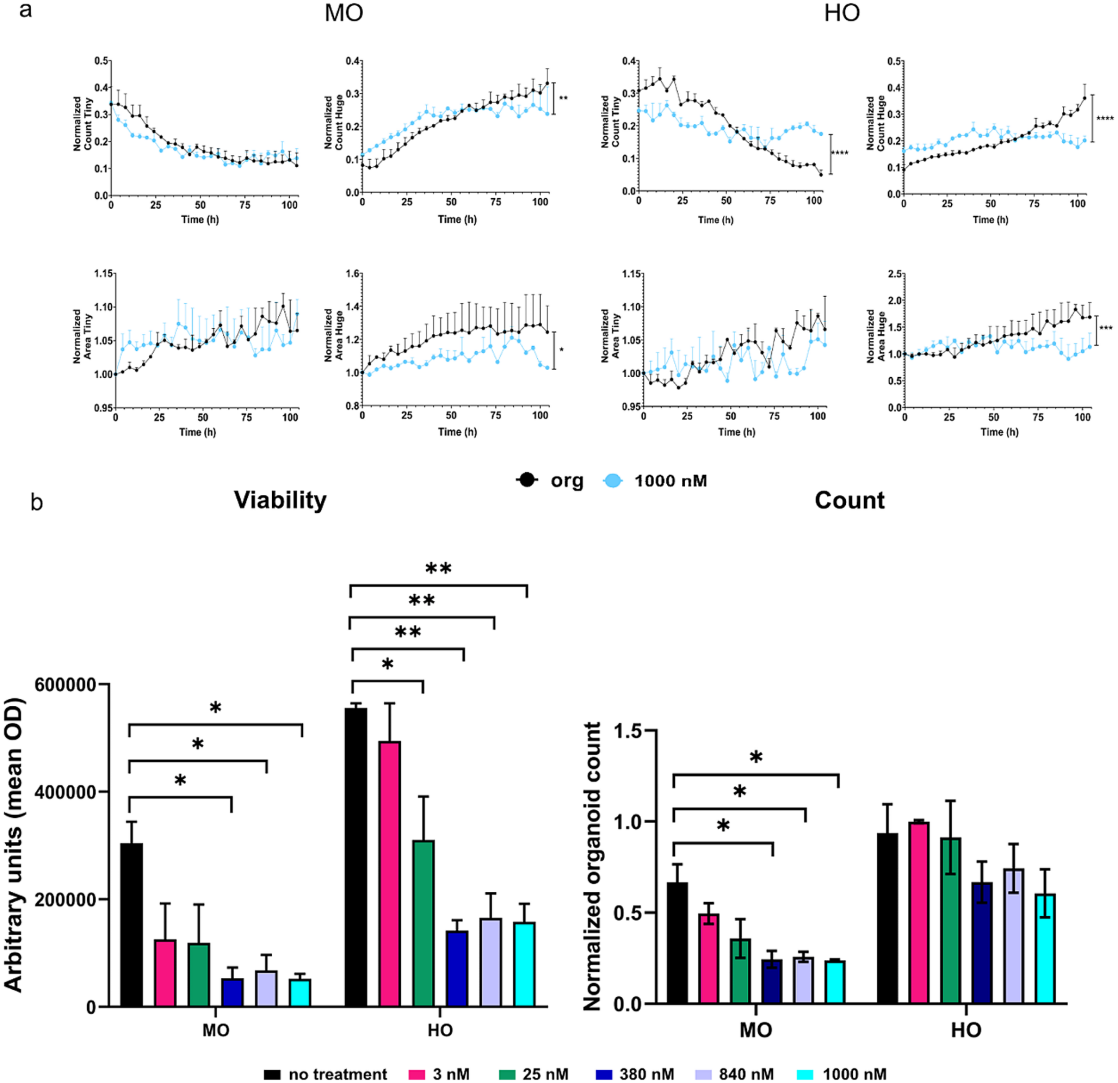
Size-dependent response of PDAC organoids to gemcitabine treatment and validation of OrganoIDNet by CellTiter-Glo. (**a**) OrganoIDNet effectively clustered PDAC organoids based on size, here “Huge” and “Tiny”, and stimulation with 1000 nM gemcitabine specifically targeted the Huge mouse (MO, left) and human (HO, right) PDAC organoids. Raw data were normalized by the respective baseline control. (**b**) CellTiter-Glo assay was conducted following a 100 h treatment of organoids with gemcitabine at different concentrations (3 nM to 1000 nM). Viability of organoids was quantified as optical density (OD) from ATP levels for each condition normalized to untreated mouse or human organoids (left). For comparison, the right graph shows the organoid count calculated by OrganoIDNet at the same time point in response to gemcitabine. Counts in (**a**) were normalized to initial time points and data are presented as mean ± SEM of two biological samples, with two technical replicates each. Statistical significance is indicated as follows: * *p* < 0.05, ** *p* < 0.01, *** *p* < 0.001, **** *p* < 0.0001 (Two-way ANOVA followed by Dunnett’s comparisons for (a); One-way ANOVA followed by Dunnett’s comparisons test in (b))

Despite the attempt to reduce the heterogeneity of organoids by seeding an exact number of single cells to make the organoids on day zero, after 3 days different sizes of organoids developed. To break down this heterogeneity for analysis, we clustered the organoids into five different size bins: Tiny, Small, Medium, Large, Huge and evaluated their growth pattern during the treatment (supplementary Fig. S1). Interestingly, we observed a size-dependent response to gemcitabine, with an increase in the amount of Tiny organoids along with a decrease in the number of Huge organoids (Fig. 5a). This effect was more pronounced in the human organoids, with a gemcitabine concentration dependent change, reaching about a 50% decrease for Huge organoids in response to 1000 nM gemcitabine treatment in comparison to untreated cells and a parallel increase in Tiny organoids (Fig. 5a, right panel; supplementary Fig. S1) (supplementary VIDEO S3). This underlines the strength of this AI-based novel analytic platform to identify size-based differences in organoid response to different concentrations of gemcitabine.

To validate the OrganoIDNet platform data analysis, we employed CellTiter-Glo, a widely used assay to evaluate organoid viability, as an endpoint measurement. We observed a significant decrease in both murine and human organoid viability after 104 h, at gemcitabine concentrations of 380 nM and above in comparison to controls (Fig. 5b, left graph), which is in line with a decreased organoid count and a reduced mean area calculated by the algorithm at the last time point (Figs. 3 and 5b, right graph).

### Assessment of organoid/PBMCs co-cultures in response to Atezolizumab using OrganoidIDNet

**Fig. 6:**
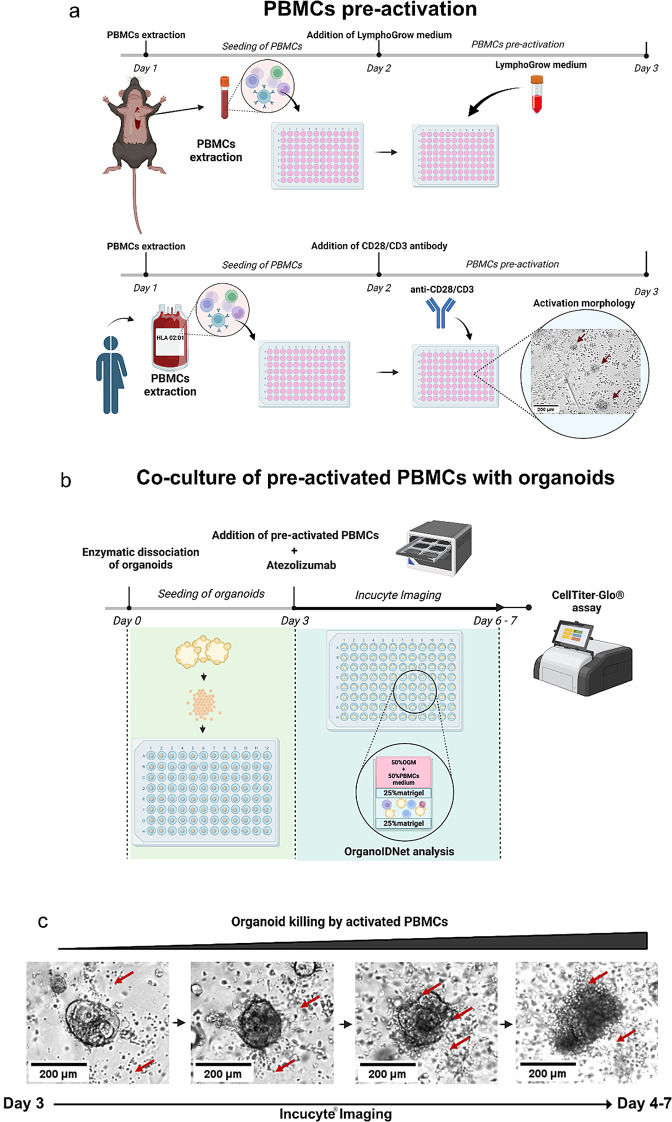
Methodological overview for evaluating the impact of Atezolizumab on organoid/ PBMC co-cultures (**a**) Schematic representation of PBMC pre-activation in the presence of LymphoGrow Medium or anti-CD28/CD3 antibody for mouse and human PBMCs, respectively, to facilitate PBMC activation. PBMC activation was confirmed by live-cell imaging using the Incucyte system by demonstration of aggregation of PBMCs (red arrows). (**b**) Schematic illustration showing the sandwich protocol that facilitates the direct interaction between PBMCs and PDAC organoids, while ensuring a stable Z-position for imaging with the Incucyte. On day 3, pre-stimulated PBMCs and fully-grown PDAC organoids were co-cultured and subjected to Atezolizumab stimulation. Incucyte images were captured over 3–4 days and analyzed using the OrganoIDNet algorithm. Organoid viability was assessed at the end of the experiment using a luminescent CellTiter-Glo assay. (**c**) Representative bright-field images depicting the patient-derived organoid cytotoxicity elicited by stimulated human PBMCs (marked by red arrows) from days 3–7. Scale bars: 200 µM

Atezolizumab, a PD-L1 checkpoint inhibitor, has emerged as a promising immunotherapeutic strategy for cancer treatment [44]. In PDAC, the antibody has been shown to potentiate the immune response of a personalized mRNA vaccine [11]. As organoids lack any immune aspect, testing immunotherapeutic strategies requires the active addition of immune cells to the system [23]. We therefore devised a structured protocol involving a sandwiched co-culture of murine and human PDAC organoids with PBMCs and then evaluated the precision of OrganoIDNet in monitoring alterations in number, size, and morphology in these PDAC organoid/PBMC co-cultures following Atezolizumab treatment. For murine PDAC organoids, we collected the PBMCs from healthy mice of the same mouse strain, and for human co-cultures, we used PDAC organoids with HLA-matched PBMCs derived from healthy donors. As illustrated in Fig. 6a, the initial step involved pre-activating human and mouse PBMCs by adding ImmunoCult™ Human CD3/CD28 T Cell Activator or LymphoGrow medium, respectively. The binding of CD3/CD28 leads to a series of intracellular events which results in human T cell proliferation and the production of cytokines, contributing to an effective immune response to enable their interaction with the PDAC PDOs. This activation process in human PMBCs was observed through morphological changes, such as the formation of immune cell clumps (Fig. 6a), in accordance with the manufacturer’s observations applying the anti-CD28/CD3 antibody. Subsequently, the organoids were either cultured alone as controls or in combination with the activated immune cells, with the entire assembly enclosed between two Matrigel layers (Fig. 6b). This arrangement facilitates direct interaction between the two cell populations, preserving their spatial orientation and allowing for stable live-imaging of the co-cultures. Our pancreatic cancer/immune cell co-culture approach thus enabled mouse-derived PBMCs of the same mouse strain or HLA-matched human PBMCs to have direct contact with the respective murine and human-derived PDAC organoids for an extended period of 72–100 h. Furthermore, by trapping PDAC organoids with PBMCs between two layers of matrigel, we could assess live-imaging readouts with a stable Z-plane enabling accuracy in the image data collection. With the application of our OrganoIDNet platform, we could show with accuracy the PDAC organoid responses without destabilizing the co-cultures.

We observed the expansion of human and mouse organoids in control conditions without drug treatment, affirming that our experimental conditions did not compromise the viability of the organoids over time (supplementary VIDEO S4). Following the addition of Atezolizumab in combination with MOGM or HOGM, along with a 50% PBMCs culture medium supporting both immune cell and organoid proliferation, our analytical strategy comprised live-cell imaging acquisition through the Incucyte® system over a 4- or 3-day interval, respectively. Subsequently, a CellTiter-Glo assay was employed to evaluate the viability of PDAC organoids at the conclusion of the experiment.

The live-cell imaging data was processed using the OrganoIDNet algorithm, and the accuracy of the analysis was confirmed through a comparison with the CellTiter-Glo data (Fig. 6b). The Atezolizumab potency was evaluated by comparing co-culture read-outs (+ PBMCs + Atezolizumab) with those from organoid/PBMCs co-cultures without Atezolizumab (+ PBMCs) and organoids alone (Org). This stepwise approach allowed us to dynamically observe and analyze the real-time killing process of activated PBMCs on organoids, as represented in Fig. 6c and supplementary VIDEO S5. From day 3, at the start of the organoid/PBMCs co-culture, pre-activated PBMCs (small bright cells marked by the red arrows) started to surround the organoids and dismembering the outer layer of the organoid, ultimately leading to the disintegration of the organoid structure to a 2D clumping of tumor cells.

**Fig. 7:**
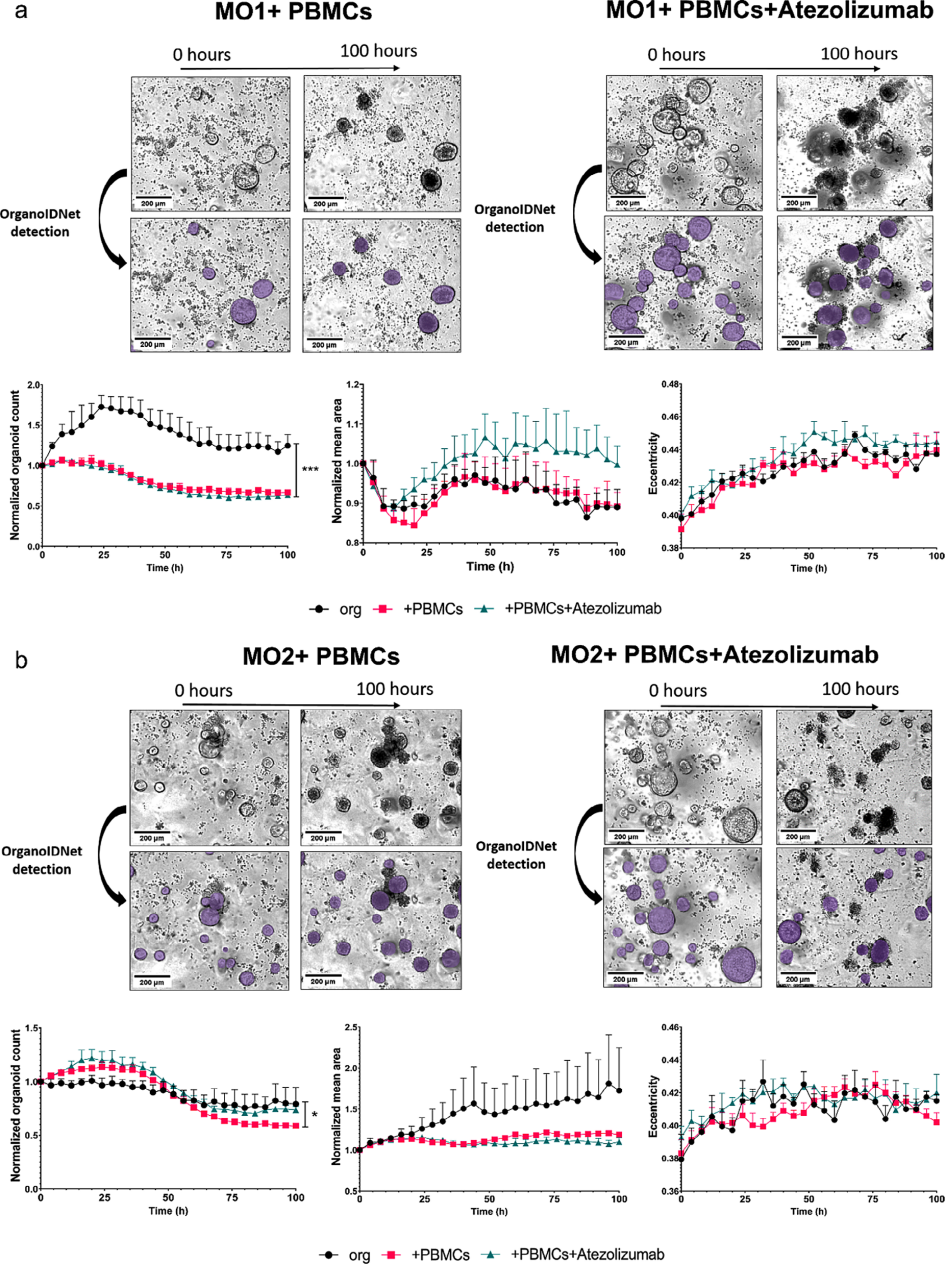
Decreased count of murine organoids in organoid/PBMC co-cultures. Representative Incucyte images and quantification of counts, area, and eccentricity by applying OrganoIDNet of MO1 (**a**) and MO2 (**b**) co-cultured with PBMCs alone or with the addition of Atezolizumab, compared to organoid-only conditions over a 100 h observation period. All parameters were normalized to the initial time point, and the data are presented as mean ± SEM of three technical replicates. * *p* < 0.05, ** *p* < 0.01, *** *p* < 0.001 (Two-way ANOVA followed by Dunnett’s comparisons). Scale bars: 200 µM

**Fig. 8:**
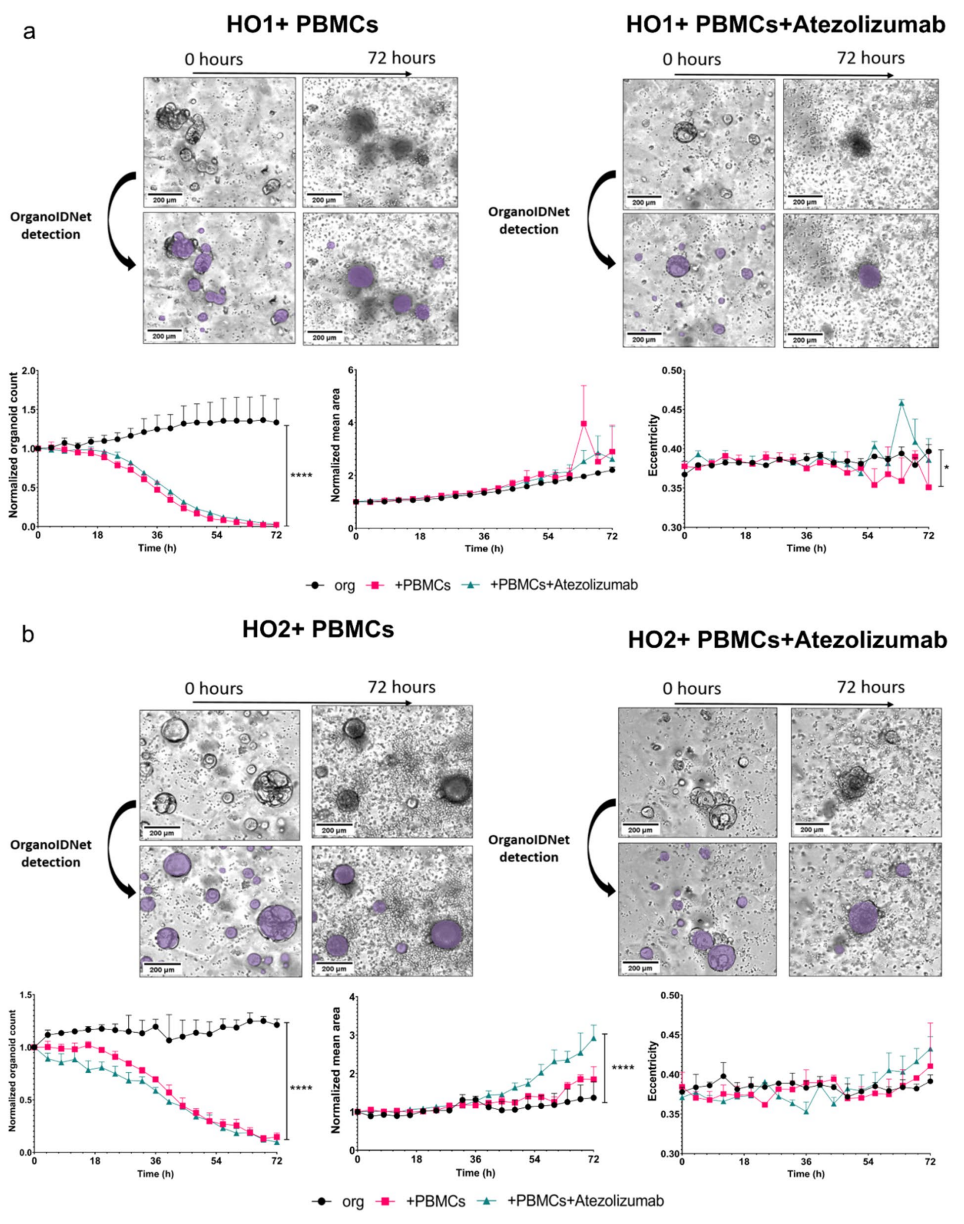
Distinct response of individual human organoids to PBMC co-cultures with Atezolizumab treatment. Representative Incucyte images and quantification of counts, area, and eccentricity applying OrganoIDNet of HO1 (**a**) and HO2 (**b**), co-cultured with PBMCs alone or with the addition of Atezolizumab, compared to organoid-only conditions as controls over a 72 h observation period. All parameters were standardized to the initial time point, and the findings are presented as mean ± SEM of three technical replicates. Statistical significance is denoted as follows: * *p* < 0.05, **** *p* < 0.0001 (Two-way ANOVA followed by Dunnett’s comparisons). Scale bars: 200 µM

Co-cultures of both murine or human organoids with PBMCs only or treated with Atezolizumab in addition to PBMCs led to a general decrease in the count of organoids (Figs. 7a and b and 8a and b, left graphs). Regarding the parameter mean area the individual organoids reacted distinctly to the addition of PBMCs and immunotherapy. MO1 showed a higher mean area over time with the combination of PBMCs and Atezolizumab, albeit this difference was not significant compared to organoids alone or the organoid/PBMC co-cultures, while MO2 mean area was not influenced by the addition of PMBCs or PBCMs + Atezolizumab (Fig. 7a, b, middle graphs). For human organoids, mean area was significantly elevated with the addition of Atezolizumab when compared to organoids alone or the addition of PBMCs, but only for HO2 (Fig. 8a, b middle graphs). Eccentricity did not substantially change with any condition in both murine and human organoids (Fig. 7a, b, and Fig. 8a, b, right graphs).

**Fig. 9:**
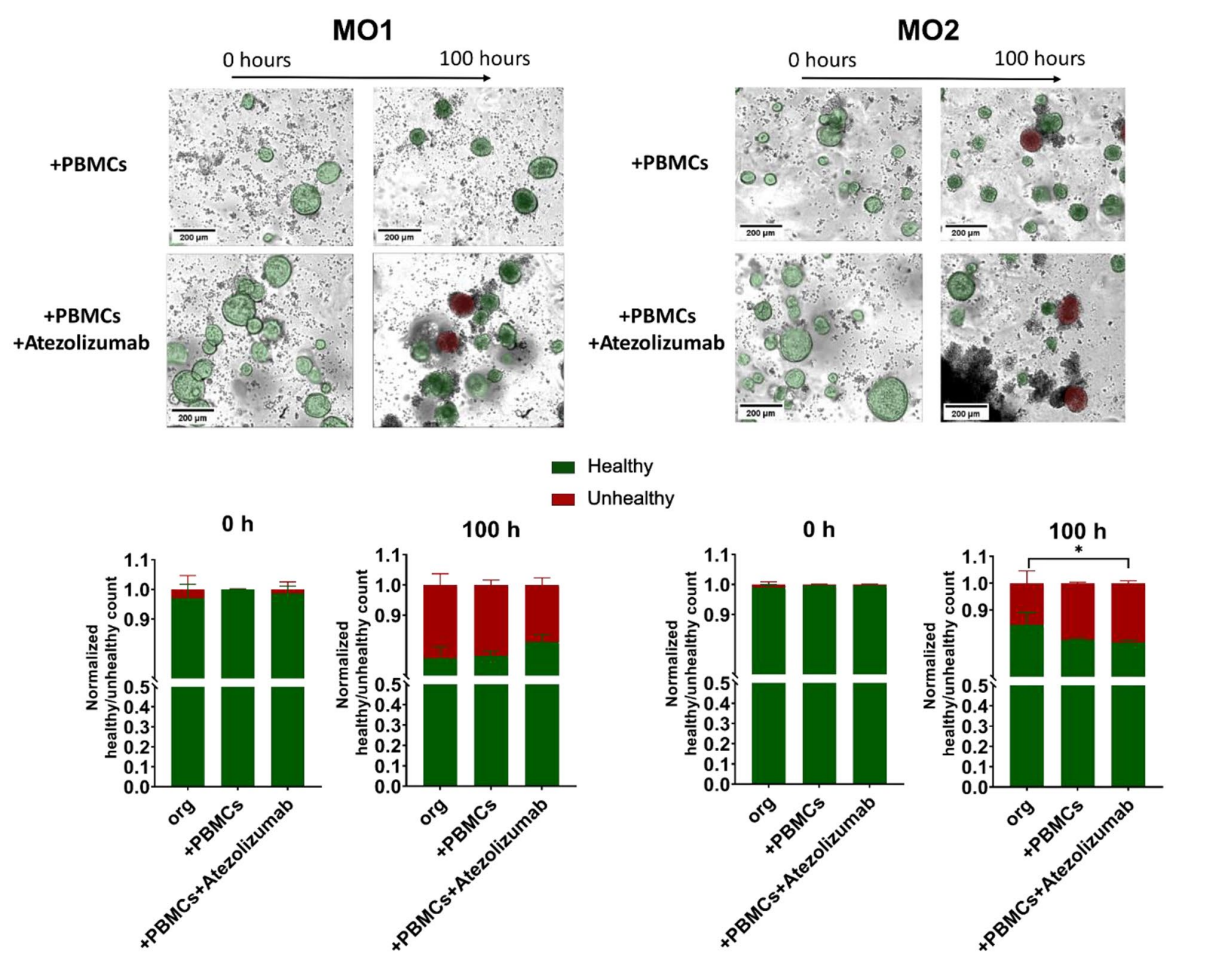
Distinct response of mouse organoid/PBMC co-cultures to Atezolizumab treatment. Representative images and quantification of unhealthy (red) and healthy (green) organoids by applying OrganoIDNet for MO1 (left panel) and for MO2 (right panel) in co-cultures with PBMCs alone or with the addition of Atezolizumab (at 0 h in comparison to 100 h). Counts were normalized to the initial time point, and the data are presented as mean ± SEM of three technical replicates (lower panel). Statistical significance is indicated as follows: * *p* < 0.05 (Two-way ANOVA followed by Dunnett’s comparisons)

**Fig. 10:**
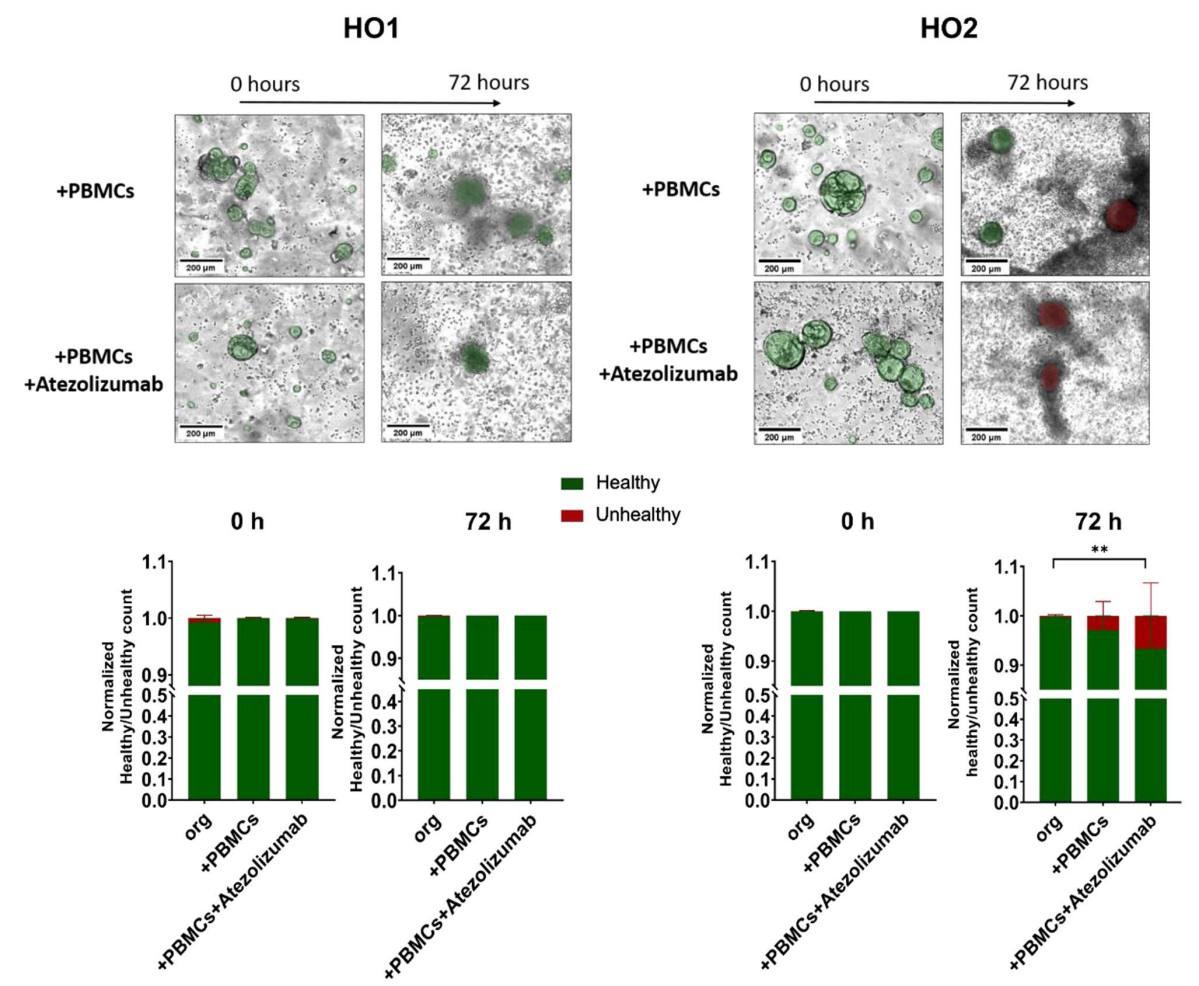
Distinct response of human organoid/PBMC co-cultures to Atezolizumab treatment. Representative images and quantification of unhealthy (red) and healthy (green) organoids by applying OrganoIDNet for HO1 (left panel) and for HO2 (right panel) in co-cultures with PBMCs alone or with the addition of Atezolizumab. All parameters were standardized to the initial time point, and the findings are presented as mean ± SEM of three technical replicates. Statistical significance is indicated as follows: ** *p* < 0.01; Scale bars: 200 µM

An organoid-individual response was also detectable in the healthy/unhealthy ratio of both mouse and human organoids. MO1 and HO1 showed no significant difference in the amount of unhealthy cells between the 3 conditions (organoids alone, organoids + PBMCs and organoids + PBMCs + Atezolizumab) (Figs. 9 and 10, left panels). However, both MO2 and HO2 showed a slightly higher ratio of unhealthy cells in response to Atezolizumab. While the addition of PBMCs to organoids MO2 and HO2 already led to a higher amount of unhealthy cells compared to organoids alone, the additional incubation with Atezolizumab further increased the unhealthy ratio, leading to a significant difference between organoids alone and organoid/PBMC/Atezolizumab co-cultures (Figs. 9 and 10, right panels).

Further analysis of organoids of different sizes revealed no significant alterations in individual murine PDAC organoids (supplementary Fig. S2a). Conversely, huge-sized HO2 organoids appeared to benefit from the addition of Atezolizumab to the medium, while no size-dependent differences were observed in HO1 (supplementary Fig. S2b).

**Fig. 11:**
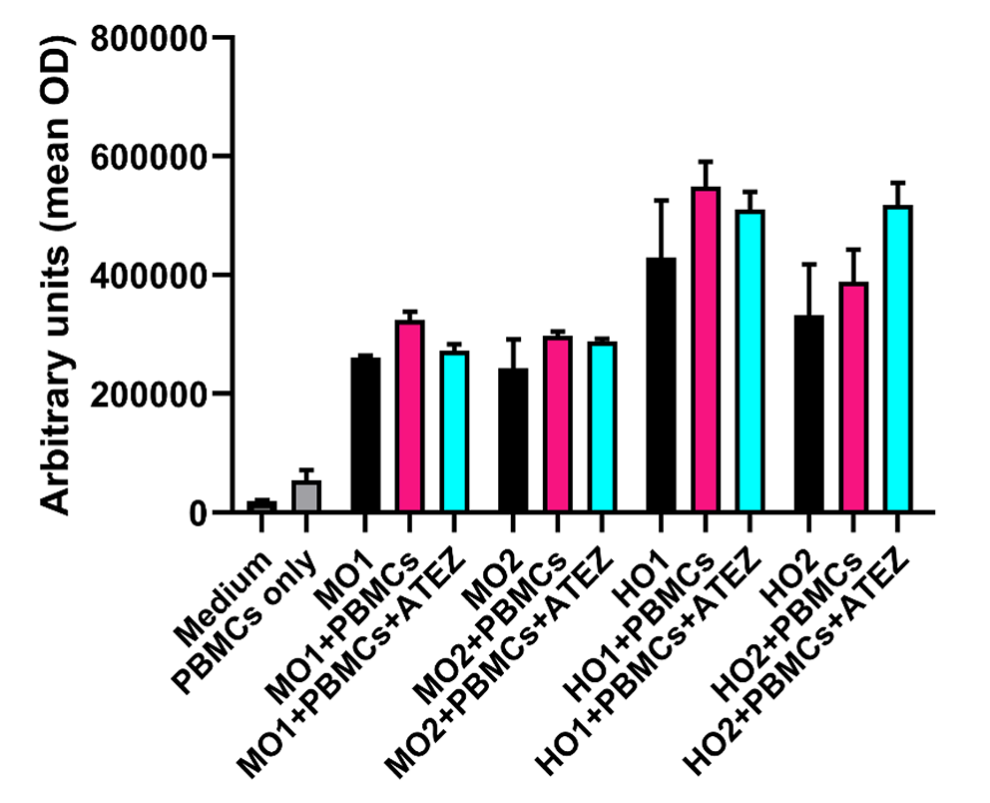
CellTiter-Glo viability assay does not reflect OrganoIDNet results of co-coltures. Mean OD is shown after a 100 h (murine) or 72 h (human) co-cultivation of individual murine (MO1 and MO2) and human (HO1 and HO2) PDAC organoids with their respective pre-activated mouse and human PBMCs (+ PBMCs) or with the addition of Atezolizumab (+ PBMCs + Atezolizumab). Findings are presented as mean ± SEM of two technical replicates

Comparison of the results obtained with OrganoIDNet with the standard viability assay CellTiter-Glo assay at the end of the co-culture experiments (100 h for mouse, 72 h for human co-cultures) (Fig. 11), surprisingly indicated that even with the addition of PBMCs or PBMCs + Atezolizumab, the viability values did not change in comparison to organoids alone, for both mouse and human organoids, showing that an endpoint assay provides inaccurate results.

**Fig. 12:**
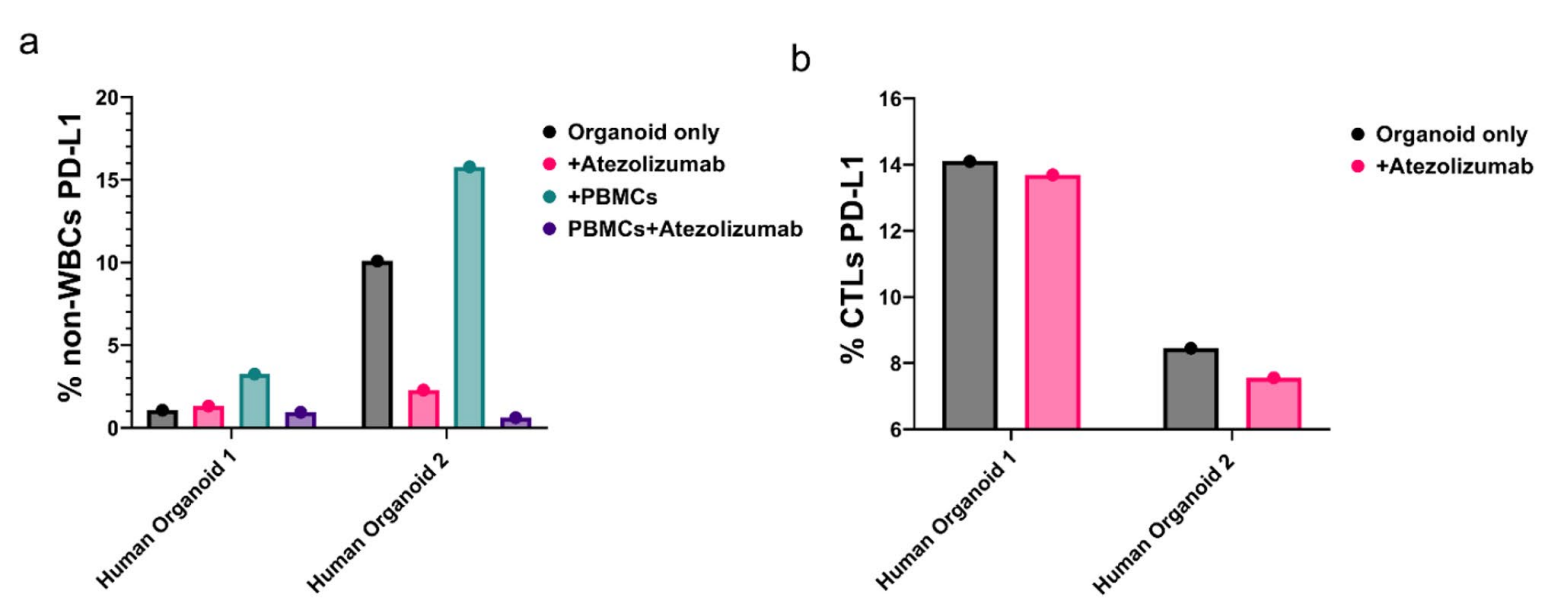
Atezolizumab efficacy may be dependent on PD-L1 expression of organoids. Baseline PD-L1 was higher in HO2 and Atezolizumab decreased the levels of PD-L1 expression in human PDAC organoids and in human CTLs cells. (**a**) At endpoint of the organoid/PBMCs co-cultures (72 h), PD-L1 expression levels were evaluated from non-white blood cells (%non-WBCs) (left graph) which represented the tumor cells from HO1 and HO2 alone (organoid only and + Atezolizumab conditions) and from the co-cultures (+ PBMCs and + PBMCs + Atezolizumab). (**b**) Cells from the PBMCs were gated to identify the cytotoxic T lymphocytes (CTLs), and percentage of PD-L1 expressing cells (%CTLs PD-L1) was quantified from the co-cultures (+ PBMCs and + PBMCs + Atezolizumab conditions). Findings are presented as mean ± SEM of two biological replicates

To investigate whether the higher sensitivity of HO2 to Atezolizumab was attributed to differing PD-L1 expression levels, we conducted flow cytometry for PD-L1 quantification on human organoids cultured alone, with or without Atezolizumab, and in combination with PBMCs, with or without Atezolizumab (Fig. 12). As expected, the addition of Atezolizumab resulted in a decrease in PD-L1 levels in tumor cells of both human organoids and cytotoxic T-lymphocytes (CTLs). As the baseline PD-L1 expression in tumor cells of HO1 was notably lower than the levels of PD-L1 in HO2, we speculate that PD-L1 expression may indeed play a role in the Atezolizumab effect on PDAC organoid response. These findings underscore the efficacy of OrganoIDNet in discerning personalized organoid responses to Atezolizumab treatment.

## Discussion

Here we provide an organoid-imaging-based platform including the image-based optimization of organoid/PBMCs co-culture for the evaluation of organoid parameters such as count, area and eccentricity over time and in response to chemo- and immunotherapy. For this we set up an image-stable organoid/immune cell co-culture and developed OrganoIDNet, a deep learning -based algorithm capable of analyzing bright-field (BF) images over time. We show that this platform is able to accurately predict and real-time monitor not only the response of both murine and human PDAC organoids to gemcitabine, a standard chemotherapy for PDAC treatment, but also to assess the response to PD-L1 inhibition as immunotherapy in PDAC organoid/immune cell co-cultures. OrganoIDNet allows us to stratify organoids into distinct size categories and assess their behavior towards the treatment. We observed a concentration-dependent decrease in organoid count and area in response to gemcitabine, affecting particularly the larger-sized organoids. This highlights the potential of this analytic tool to predict size-specific drug responses.

Moreover, in the untreated organoids we observed a shift in organoid morphology towards a more elongated shape during organoid expansion over time, which did not occur anymore upon gemcitabine treatment. An elongated shape of organoids has been associated with a better capacity to invade the Matrigel indicating a tendency to epithelial to mesenchymal transition (EMT). This may indicate that the organoids used here are not resistant to gemcitabine as they did not change their more round morphology in the presence of chemotherapy. A similar effect has been described already for some chemotherapeutics in mammary tumor organoids [45].

In addition, OrganoIDNet enabled the distinction between unhealthy and healthy organoids based on the dark intensity of the spots of the unhealthy organoids [32]. This was possible due to the acquisition of brightfield microscopy images (as opposed to fluorescence microscopy), which presented an advantage for both the algorithm’s development and the maintenance of a physiological sample environment that does not require fluorescent dyes for organoid staining. Problems arising from the latter, such as photobleaching and phototoxicity, have been discussed by Fei et al. [46]. BF images were thus ideal for the segmentation of healthy/unhealthy organoids with the OrganoIDNet algorithm, whose accuracy was verified by us with the endpoint-viability protocol CellTiter-Glo used as a standard measure for treatment efficacy. Although with the expansion of AI in research, several algorithms have emerged for organoid analysis [47–51], our algorithm is the first deep-learning algorithm trained with a dataset from both murine and human matrigel-free organoid co-cultures with immune cells. This provided information on organoid growth kinetics and morphology from more complex organoid conditions, such as co-cultures. Furthermore, with our custom-trained StarDist model [34] with manually annotated organoid identification in those complex conditions, we achieved a better segmentation for both sparsely and densely populated organoid images than previous studies [47–51]. Importantly, tracking entire regions of interest instead of tracking single organoids, as has been reported before [47], enabled OrganoIDNet to accurately capture organoid growth behavior, such as the merging of two organoids. Such processes could be wrongly interpreted as organoid death when using single organoid tracking, whereas our algorithm correctly interprets the merge as the growth or expansion of organoids. Assessing variability of organoids is particularly valuable for personalized medicine, as it mirrors the natural diversity found in human tissues. These intrinsic organoid traits provide researchers with a model encompassing various genetic backgrounds and reflecting patient tumor heterogeneity. Moreover, the inter- and intra-patient variability can aid in modeling patient-specific responses, potentially leading to more tailored treatments. OrganoIDNet provides a platform capable of discerning specific size responses while preserving such variability inherent to organoid cultures. However, it is important to note that organoids from different tissue sources or tumor entities may produce different shapes, which requires additional training of OrganoIDNet.

Following the demonstration of the capacity of OrganoIDNet to detect the response to gemcitabine, a currently used PDAC chemotherapeutic drug, we then applied our platform to monitor the efficacy of the PD-L1 inhibitor Atezolizumab in PDAC organoid and PBMC co-cultures. For this purpose, we established a co-culture protocol based on the Triple-Decker sandwich method by Cambra et al. [52]. Here we show that this technique ensures the entrapment of organoids and PBMCs in the same Z-position, with direct contact between the two populations, and thus enables more robust image acquisition of the co-cultures. This is a step forward in relation to previous studies [47–51] by increasing the time of the co-cultures in an optimized protocol that allows live imaging acquisition. Moreover, although CellTiter-Glo is the current standard protocol to assess organoid viability, we show that this assay reveals its limitations in an organoid co-culture set-up. We found no differences in CellTiter-Glo-assessed viability between treated and untreated groups. At first sight this result may seem in stark contrast to the drastically reduced organoid count that OrganoIDNet detected in response to PBMCs or PBMCs + Atezolizumab. An explanation for this result may be, however, that while organoids that were co-cultured with PBMCs or PBMCs + Atezolizumab disintegrated and were thus not recognized and counted by OrganoIDNet, the dissociated cells were still viable and thus detectable by CellTiter-Glo. Using this assay we observed a trend towards a higher viability with the addition of PBMCs or PBMCs + Atezolizumab, albeit the difference to organoids alone was not significant. This difference is most likely due to the additional cells that are present in the co-cultures with the addition of PBMCs, as PBMCs alone in culture already lead to a higher viability when compared to medium alone. The CellTiter-Glo result of co-cultures thus revealed that a viability assay is not an adequate method for providing sufficient information on the status of organoids over time and may skew the result towards a more “healthy” status when in fact the number of intact organoids has drastically decreased as visualized by live cell imaging. Moreover, CellTiter-Glo relies on luminescent quantification of intracellular ATP, which can be affected by inter-patient growth rate variations and metabolic modulations induced by drugs. Additionally, it is limited to a single time-point analysis and cannot determine the mechanistic action of the drug (cytostatic or cytotoxic response) over time.

The implementation of the OrganoIDNet analysis in our optimized live-imaging protocol allowed for real-time monitoring of organoid responses to Atezolizumab, providing individual read out not only on the number but also on the general organoid morphological features, such as organoid count, sizes, area and eccentricity. More importantly, this approach also provides the healthy/unhealthy status of the organoid cultures showing individual treatment responses of organoids. Specifically, Atezolizumab potentiated the toxicity of the PBMCs on HO2 by increasing the number of unhealthy organoids. This prompted us to hypothesize whether the distinct mutational landscape, marked by the different mutations in *KRAS*, *SMAD4*, *TP53* and *CDKN2A* (Table 2) could explain the different responses of the two human PDAC organoids to Atezolizumab stimulation, as studies are trying to understand the tumor heterogeneity in the response of patients to Immune Checkpoint Inhibitors (ICIs) treatment [53]. This result supports numerous reports about the individual response of patients to therapy and shows the advantages of human organoid/immune cell co-cultures derived from patients as a model for personalized drug testing. While traditional clinical methods for therapy decisions, such as molecular profiling, imaging, or histopathological analysis, can currently not be replaced by organoid based approaches, organoid testing could be integrated with these established methods, in particular for tumor entities with heterogeneous therapeutic response [54]. Our platform demonstrates that the integration of artificial intelligence with real-time imaging of organoids and immune co-cultures can improve preclinical and clinical evaluations of cancer immunotherapeutics and may thus support precision medicine.

## Conclusion

We are presenting a comprehensive platform for the characterization and monitoring of organoids alone or in co-culture with immune cells in response to treatment. The utilization of our developed OrganoIDNet algorithm enables not only the accurate quantification of important parameters of organoid cultures, such as count, mean area, and eccentricity over time but also allows a stratified assessment of organoid response to treatment in dependence of size. Our advanced organoid/PBMC co-culture protocol, which incorporates the important aspect of immune cells for the evaluation of immunotherapies, vividly demonstrates the power of OrganoIDNet to use PDOs for personalized pharmaco-phenotyping for the advancement of therapeutic strategies.

## Electronic supplementary material

Below is the link to the electronic supplementary material.

Supplementary Material 1

Supplementary Material 2

Supplementary Material 3

Supplementary Material 4

Supplementary Material 5

Supplementary Material 6

## Data Availability

Imaging data used for OrganoIDNet training have been deposited at https://zenodo.org/records/10643410.

## References

[1] J.X. Hu, C.F. Zhao, W.B. Chen, Q.C. Liu, Q.W. Li, Y.Y. Lin et al., Pancreatic cancer: a review of epidemiology, trend, and risk factors. World J. Gastroenterol. **27**(27), 4298–4321 (2021) 34366606 10.3748/wjg.v27.i27.4298 PMC8316912

[2] A. Adamska, A. Domenichini, M. Falasca, Pancreatic ductal adenocarcinoma: current and evolving therapies. Int. J. Mol. Sci. **18**(7), 1338 (2017) 28640192 10.3390/ijms18071338 PMC5535831

[3] R.L. Siegel, K.D. Miller, A. Jemal, Cancer statistics, 2018. CA Cancer J Clin. 2018;68(1):7–30 10.3322/caac.21442 29313949

[4] A.K. Witkiewicz, E.A. McMillan, U. Balaji, G. Baek, W.C. Lin, J. Mansour et al., Whole-exome sequencing of pancreatic cancer defines genetic diversity and therapeutic targets. Nat. Commun. **6**, 6744 (2015) 25855536 10.1038/ncomms7744 PMC4403382

[5] E.S. Christenson, E. Jaffee, N.S. Azad, Current and emerging therapies for patients with advanced pancreatic ductal adenocarcinoma: a bright future. Lancet Oncol. **21**(3), e135–e145 (2020) 32135117 10.1016/S1470-2045(19)30795-8 PMC8011058

[6] J.A. Seidel, A. Otsuka, K. Kabashima, Anti-PD-1 and Anti-CTLA-4 therapies in Cancer: mechanisms of Action, Efficacy, and limitations. Front. Oncol. **8**, 86 (2018) 29644214 10.3389/fonc.2018.00086 PMC5883082

[7] E. Karamitopoulou, Tumour microenvironment of pancreatic cancer: immune landscape is dictated by molecular and histopathological features. Br. J. Cancer. **121**(1), 5–14 (2019) 31110329 10.1038/s41416-019-0479-5 PMC6738327

[8] J.R. Brahmer, S.S. Tykodi, L.Q.M. Chow, W.J. Hwu, S.L. Topalian, P. Hwu et al., Safety and activity of anti-PD-L1 antibody in patients with advanced cancer. N Engl. J. Med. **366**(26), 2455–2465 (2012) 22658128 10.1056/NEJMoa1200694 PMC3563263

[9] G. Sonpavde, PD-1 and PD-L1 inhibitors as salvage therapy for Urothelial Carcinoma. N Engl. J. Med. **376**(11), 1073–1074 (2017) 28212061 10.1056/NEJMe1701182

[10] T. Nomi, M. Sho, T. Akahori, K. Hamada, A. Kubo, H. Kanehiro et al., Clinical significance and therapeutic potential of the programmed death-1 ligand/programmed death-1 pathway in human pancreatic cancer. Clin. Cancer Res. **13**(7), 2151–2157 (2007) 17404099 10.1158/1078-0432.CCR-06-2746

[11] L.A. Rojas, Z. Sethna, K.C. Soares, C. Olcese, N. Pang, E. Patterson et al., Personalized RNA neoantigen vaccines stimulate T cells in pancreatic cancer. Nature. **618**(7963), 144–150 (2023) 37165196 10.1038/s41586-023-06063-y PMC10171177

[12] D. Arnold, M. Collienne, A. Stein, G. Ungerechts, E. Goekkurt, J. Chater et al., 650 pelareorep combined with atezolizumab and chemotherapy demonstrates encouraging results as first-line treatment in advanced or metastatic pancreatic ductal adenocarcinoma (PDAC) patients– interim results from the GOBLET study

[13] X. Zhou, Y. Ni, X. Liang, Y. Lin, B. An, X. He et al., Mechanisms of tumor resistance to immune checkpoint blockade and combination strategies to overcome resistance. Front. Immunol. **13**, 915094 (2022) 36189283 10.3389/fimmu.2022.915094 PMC9520263

[14] C. Su, K.A. Olsen, C.E. Bond, V.L.J. Whitehall, The efficacy of using patient-derived organoids to predict treatment response in Colorectal Cancer. Cancers (Basel). **15**(3), 805 (2023) 36765763 10.3390/cancers15030805 PMC9913532

[15] P.W. Nagle, J.T.M. Plukker, C.T. Muijs, van P. Luijk, R.P. Coppes, Patient-derived tumor organoids for prediction of cancer treatment response. Semin Cancer Biol. **53**, 258–264 (2018) 29966678 10.1016/j.semcancer.2018.06.005

[16] L. Demyan, A.N. Habowski, D. Plenker, D.A. King, O.J. Standring, C. Tsang et al., Pancreatic Cancer patient-derived Organoids can predict response to Neoadjuvant Chemotherapy. Ann. Surg. **276**(3), 450–462 (2022) 35972511 10.1097/SLA.0000000000005558 PMC10202108

[17] H.M. Wang, C.Y. Zhang, K.C. Peng, Z.X. Chen, J.W. Su, Y.F. Li et al., Using patient-derived organoids to predict locally advanced or metastatic lung cancer tumor response: a real-world study. Cell. Rep. Med. **4**(2), 100911 (2023) 36657446 10.1016/j.xcrm.2022.100911 PMC9975107

[18] G.E. Wensink, S.G. Elias, J. Mullenders, M. Koopman, S.F. Boj, O.W. Kranenburg et al., Patient-derived organoids as a predictive biomarker for treatment response in cancer patients. npj Precis Onc. **5**(1), 1–13 (2021) 10.1038/s41698-021-00168-1 PMC8042051 33846504

[19] I.R. Calvo, C. Weber, M. Ray, M. Brown, K. Kirby, R.K. Nandi et al., Human organoids Share Structural and genetic features with primary pancreatic adenocarcinoma tumors. Mol. Cancer Res. **17**(1), 70–83 (2019) 30171177 10.1158/1541-7786.MCR-18-0531 PMC6647028

[20] T.G. Krieger, Le S. Blanc, J. Jabs, F.W. Ten, N. Ishaque, K. Jechow et al., Single-cell analysis of patient-derived PDAC organoids reveals cell state heterogeneity and a conserved developmental hierarchy. Nat. Commun. **12**, 5826 (2021) 34611171 10.1038/s41467-021-26059-4 PMC8492851

[21] E. Driehuis, van A. Hoeck, K. Moore, S. Kolders, H.E. Francies, M.C. Gulersonmez et al., Pancreatic cancer organoids recapitulate disease and allow personalized drug screening. Proceedings of the National Academy of Sciences. 2019;116(52):26580–90 10.1073/pnas.1911273116 PMC6936689 31818951

[22] L. Huang, A. Holtzinger, I. Jagan, M. BeGora, I. Lohse, N. Ngai et al., Ductal pancreatic cancer modeling and drug screening using human pluripotent stem cell and patient-derived tumor organoids. Nat. Med. **21**(11), 1364–1371 (2015) 26501191 10.1038/nm.3973 PMC4753163

[23] Y. Huang, Z. Huang, Z. Tang, Y. Chen, M. Huang, H. Liu et al., Research Progress, Challenges, and Breakthroughs of Organoids as Disease Models. Frontiers in Cell and Developmental Biology [Internet]. 2021 [cited 2023 Oct 7];9. https://www.frontiersin.org/articles/10.3389/fcell.2021.740574 10.3389/fcell.2021.740574 PMC8635113 34869324

[24] L. Magré, M.M.A. Verstegen, S. Buschow, L.J.W. van der Laan, M. Peppelenbosch, J. Desai, Emerging organoid-immune co-culture models for cancer research: from oncoimmunology to personalized immunotherapies. J. Immunother Cancer. **11**(5), e006290 (2023) 37220953 10.1136/jitc-2022-006290 PMC10231025

[25] L. Holokai, J. Chakrabarti, J. Lundy, D. Croagh, P. Adhikary, S.S. Richards et al., Murine- and Human-Derived Autologous Organoid/Immune Cell Co-Cultures as Pre-Clinical Models of Pancreatic Ductal Adenocarcinoma. Cancers [Internet]. 2020 Dec [cited 2023 Oct 20];12(12). https://www.ncbi.nlm.nih.gov/pmc/articles/PMC7766822/10.3390/cancers12123816 PMC7766822 33348809

[26] M. Knoblauch, T. Ma, I. Beirith, D. Koch, F. Hofmann, K. Heinrich et al., In-vitro model to mimic T cell subset change in human PDAC organoid co-culture. J Cancer Res Clin Oncol [Internet]. 2023 Jul 20 [cited 2023 Oct 18]; 10.1007/s00432-023-05100-7 10.1007/s00432-023-05100-7 PMC10587248 37470855

[27] K.K. Dijkstra, C.M. Cattaneo, F. Weeber, M. van de Chalabi, L.F. Fanchi et al., Generation of Tumor-reactive T cells by co-culture of Peripheral Blood lymphocytes and Tumor Organoids. Cell. **174**(6), 1586–1598e12 (2018) 30100188 10.1016/j.cell.2018.07.009 PMC6558289

[28] P.O. Frappart, T.G. Hofmann, Pancreatic ductal adenocarcinoma (PDAC) organoids: the shining light at the end of the tunnel for drug response prediction and Personalized Medicine. Cancers. **12**(10), 2750 (2020) 32987786 10.3390/cancers12102750 PMC7598647

[29] M. Ischyropoulou, K. Sabljo, L. Schneider, C.M. Niemeyer, J. Napp, C. Feldmann et al., High-load Gemcitabine Inorganic-Organic hybrid nanoparticles as an image-guided tumor-selective drug-delivery system to treat pancreatic Cancer. Adv. Mater. 2023;e2305151 10.1002/adma.202305151 37587542

[30] A. Li, J.P. Morton, Y. Ma, S.A. Karim, Y. Zhou, W.J. Faller et al., Fascin is regulated by slug, promotes progression of pancreatic cancer in mice, and is associated with patient outcomes. Gastroenterology. **146**(5), 1386–1396e1 (2014) 24462734 10.1053/j.gastro.2014.01.046 PMC4000441

[31] S.S. Wilson, M. Mayo, T. Melim, H. Knight, L. Patnaude, X. Wu et al., Optimized Culture Conditions for Improved Growth and Functional Differentiation of Mouse and Human Colon Organoids. Frontiers in Immunology [Internet]. 2021 [cited 2023 Oct 16];11. https://www.frontiersin.org/articles/10.3389/fimmu.2020.547102 10.3389/fimmu.2020.547102 PMC7906999 33643277

[32] L. Klemke, J.P. Blume, De T. Oliveira, R. Schulz-Heddergott, Preparation and Cultivation of Colonic and Small Intestinal Murine Organoids Including Analysis of Gene expression and Organoid viability. Bio Protoc. **12**(2), e4298 (2022) 35127988 10.21769/BioProtoc.4298 PMC8799662

[33] E.T. Duman, M. Sitte, K. Conrads, A. Makay, F. Ludewig, P. Ströbel et al., A single-cell strategy for the identification of intronic variants related to mis-splicing in pancreatic cancer [Internet]. bioRxiv; 2023 [cited 2023 Nov 10]. p. 2023.05.08.539836. https://www.biorxiv.org/content/10.1101/2023.05.08.539836v1 10.1093/nargab/lqae057 PMC11127633 38800828

[34] U. Schmidt, M. Weigert, C. Broaddus, G. Myers, Cell detection with Star-Convex polygons, in *Medical Image Computing and Computer Assisted Intervention– MICCAI 2018*, ed. by A.F. Frangi, J.A. Schnabel, C. Davatzikos, C. Alberola-López, G. Fichtinger (Springer International Publishing, Cham, 2018), pp. 265–273. (Lecture Notes in Computer Science)

[35] J. Yan, Y. Tai, H. Zhou, Culture of Mouse Liver Ductal Organoids. Methods Mol. Biol. **2455**, 117–129 (2022) 35212991 10.1007/978-1-0716-2128-8_11 PMC9327439

[36] S. Tanida, K. Fuji, L. Lu, T. Guyomar, B.H. Lee, A. Honigmann et al., The interplay between lumen pressure and cell proliferation determines organoid morphology in a multicellular phase field model [Internet]. bioRxiv; 2023 [cited 2023 Oct 18]. p. 2023.08.17.553655. https://www.biorxiv.org/content/10.1101/2023.08.17.553655v1

[37] R.R.J. Low, K.Y. Fung, H. Gao, A. Preaudet, L.F. Dagley, J. Yousef et al., S100 family proteins are linked to organoid morphology and EMT in pancreatic cancer. Cell. Death Differ. **30**(5), 1155–1165 (2023) 36828915 10.1038/s41418-023-01126-z PMC10154348

[38] S. Shroff, A. Rashid, H. Wang, M.H. Katz, J.L. Abbruzzese, J.B. Fleming et al., SOX9: a useful marker for pancreatic ductal lineage of pancreatic neoplasms. Hum. Pathol. **45**(3), 456–463 (2014) 24418153 10.1016/j.humpath.2013.10.008 PMC3945019

[39] R.B. Schmuck, de C.V. Carvalho-Fischer, C. Neumann, J. Pratschke, M. Bahra, Distal bile duct carcinomas and pancreatic ductal adenocarcinomas: postulating a common tumor entity. Cancer Med. **5**(1), 88–99 (2016) 26645826 10.1002/cam4.566 PMC4708893

[40] M. Zapata, C. Cohen, M.T. Siddiqui, Immunohistochemical expression of SMAD4, CK19, and CA19-9 in fine needle aspiration samples of pancreatic adenocarcinoma: utility and potential role. Cytojournal. **4**, 13 (2007) 17587453 10.1186/1742-6413-4-13 PMC1936432

[41] L. Sivapalan, H.M. Kocher, H. Ross-Adams, C. Chelala, The molecular landscape of pancreatic ductal adenocarcinoma. Pancreatology. **22**(7), 925–936 (2022) 35927150 10.1016/j.pan.2022.07.010

[42] S.M.H. Kashfi, S. Almozyan, N. Jinks, B.K. Koo, A.S. Nateri, Morphological alterations of cultured human colorectal matched tumour and healthy organoids. Oncotarget. **9**(12), 10572–10584 (2018) 29535828 10.18632/oncotarget.24279 PMC5828197

[43] C. Pleguezuelos-Manzano, J. van den Puschhof, V. Geurts, J. Beumer, H. Clevers, Establishment and culture of human intestinal organoids derived from adult stem cells. Curr. Protoc. Immunol. **130**(1), e106 (2020) 32940424 10.1002/cpim.106 PMC9285512

[44] M. Hilmi, L. Bartholin, C. Neuzillet, Immune therapies in pancreatic ductal adenocarcinoma: where are we now? World J. Gastroenterol. **24**(20), 2137–2151 (2018) 29853732 10.3748/wjg.v24.i20.2137 PMC5974576

[45] N. Zhao, R.T. Powell, X. Yuan, G. Bae, K.P. Roarty, F. Stossi et al., Morphological screening of mesenchymal mammary tumor organoids to identify drugs that reverse epithelial-mesenchymal transition. Nat. Commun. **12**(1), 4262 (2021) 34253738 10.1038/s41467-021-24545-3 PMC8275587

[46] K. Fei, J. Zhang, J. Yuan, P. Xiao, Present Application and perspectives of Organoid Imaging Technology. Bioeng. (Basel). **9**(3), 121 (2022) 10.3390/bioengineering9030121 PMC8945799 35324810

[47] J.M. Matthews, B. Schuster, S.S. Kashaf, P. Liu, R. Ben-Yishay, D. Ishay-Ronen et al., OrganoID: a versatile deep learning platform for tracking and analysis of single-organoid dynamics. PLoS Comput. Biol. **18**(11), e1010584 (2022) 36350878 10.1371/journal.pcbi.1010584 PMC9645660

[48] C. Deben, De La E.C. Hoz, M.L. Compte, Van P. Schil, J.M.H. Hendriks, P. Lauwers et al., OrBITS: label-free and time-lapse monitoring of patient derived organoids for advanced drug screening. Cell. Oncol. (Dordr). **46**(2), 299–314 (2023) 36508089 10.1007/s13402-022-00750-0 PMC10060271

[49] E. Domènech-Moreno, A. Brandt, T.T. Lemmetyinen, L. Wartiovaara, T.P. Mäkelä, S. Ollila, Tellu - an object-detector algorithm for automatic classification of intestinal organoids. Dis. Model. Mech. **16**(3), dmm049756 (2023) 36804687 10.1242/dmm.049756 PMC10067441

[50] T. Kassis, V. Hernandez-Gordillo, R. Langer, L.G. Griffith, OrgaQuant: human intestinal organoid localization and quantification using deep convolutional neural networks. Sci. Rep. **9**(1), 12479 (2019) 31462669 10.1038/s41598-019-48874-y PMC6713702

[51] N. Gritti, J.L. Lim, K. Anlaş, M. Pandya, G. Aalderink, G. Martínez-Ara et al., MOrgAna: accessible quantitative analysis of organoids with machine learning. Development. **148**(18), dev199611 (2021) 34494114 10.1242/dev.199611 PMC8451065

[52] H.M. Cambra, N.P. Tallapragada, P. Mannam, D.T. Breault, A.M. Klein, Triple-Decker sandwich cultures of Intestinal Organoids for Long-Term Live Imaging, Uniform Perturbation, and statistical sampling. Curr. Protoc. **2**(1), e330 (2022) 35030297 10.1002/cpz1.330 PMC9006308

[53] Z. Chen, J. John, J.H. Wang, Why responses to immune checkpoint inhibitors are heterogeneous in head and neck cancers: Contributions from tumor-intrinsic and host-intrinsic factors. Frontiers in Oncology [Internet]. 2022 [cited 2023 Oct 22];12. https://www.frontiersin.org/articles/10.3389/fonc.2022.995434 10.3389/fonc.2022.995434 PMC9623029 36330485

[54] S. Yang, H. Hu, H. Kung, R. Zou, Y. Dai, Y. Hu, T. Wang, T. Lv, J. Yu, F. Li, Organoids: The current status and biomedical applications. MedComm (2020). 2023;4(3):e274 10.1002/mco2.274 PMC10192887 37215622

