# Supplementary material for: OrganoIDNet: a deep learning tool for identification of therapeutic effects in PDAC organoid-PBMC co-cultures from time-resolved imaging data
Source: Cell Oncol (Dordr). 2024 May 28;48(1):101–22. doi: 10.1007/s13402-024-00958-2 (PMC11850476; doi:10.1007/s13402-024-00958-2)
Supplement: Supplementary file 6 — Supplementary Material 6 [file 13402_2024_958_MOESM6_ESM.docx]

**Supplementary Information**

**Video S1.** Bright-field imaging of human organoids showing the increase of organoid darkness over time. Masking of the organoids using OrganoIDNet, based on the mean pixel intensity, depicts the change from healthy (green) to unhealthy status (red), over a period of 100 h. Dark debris and disintegrated organoid structures were not recognized by the algorithm.

**Video S2.** OrganoidIDNet captures merging human organoid events (green), which increases their area and decreases their counts.

**Video S3**. Monitoring of size-dependent responses to gemcitabine stimulation in human PDAC organoids. Representative live-cell tracking of different organoid sizes (Tiny = blue; Small = orange; Medium = green; Large = red; Huge = purple), showing the increase in tiny organoids and the decrease in huge organoids over 100 h upon 380 nM gemcitabine stimulation.

**Video S4.** Proliferation of untreated human organoids in the optimized sandwich protocol. Bright-field real-time imaging of untreated organoids shows their normal expansion in our optimized sandwich protocol.

**Video S5**. Tracking of organoid killing in organoid/PBMC co-cultures. Representative video of live-cell imaging over 4 days depicting the death of an organoid upon incubation with stimulated PBMCs with Atezolizumab. Segmentation shows the disappearance of the OrganoIDNet mask (green) denoting unhealthy organoids.


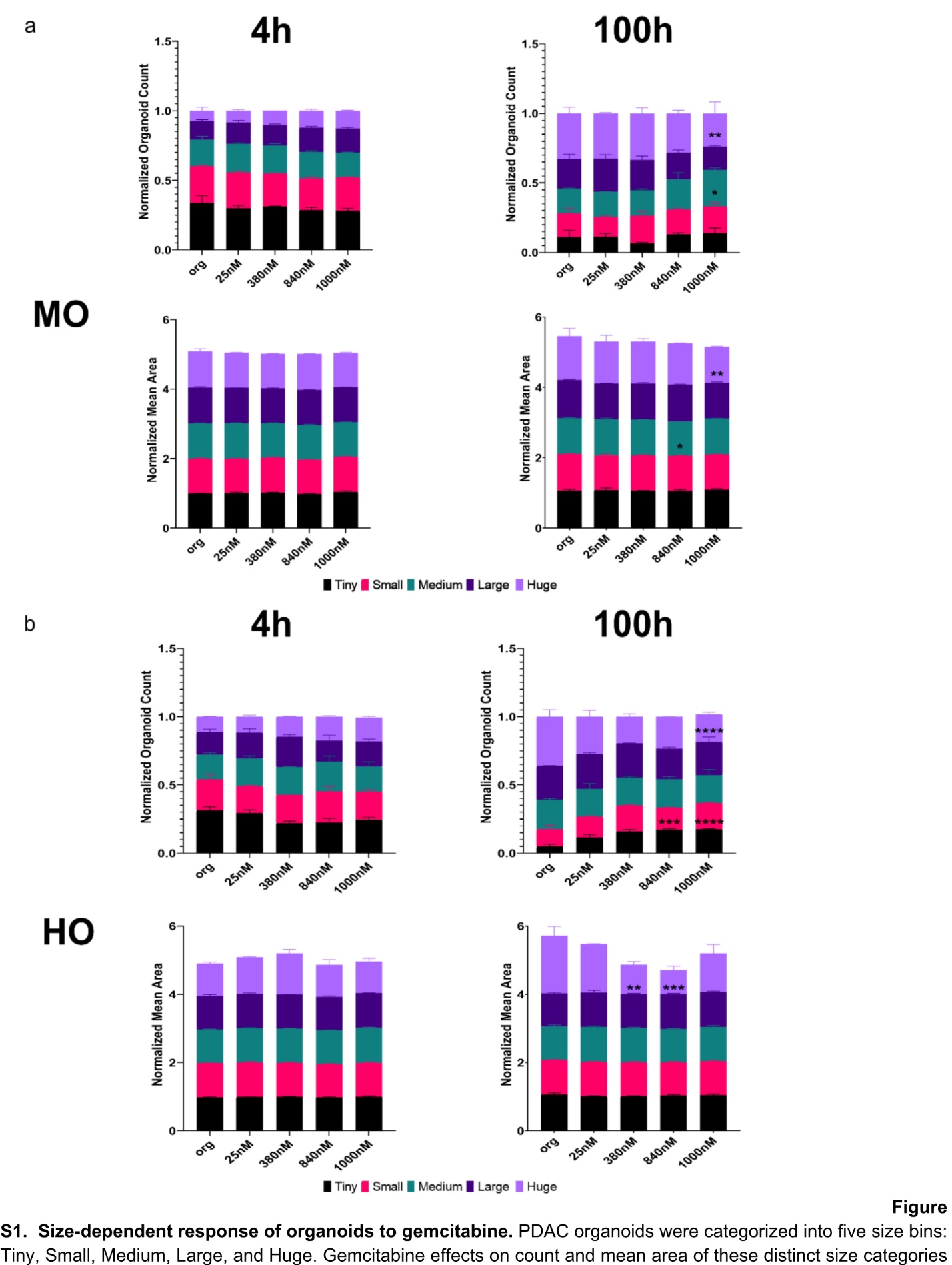


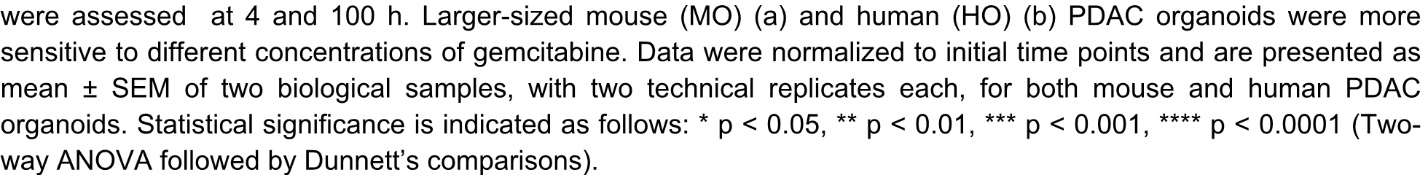


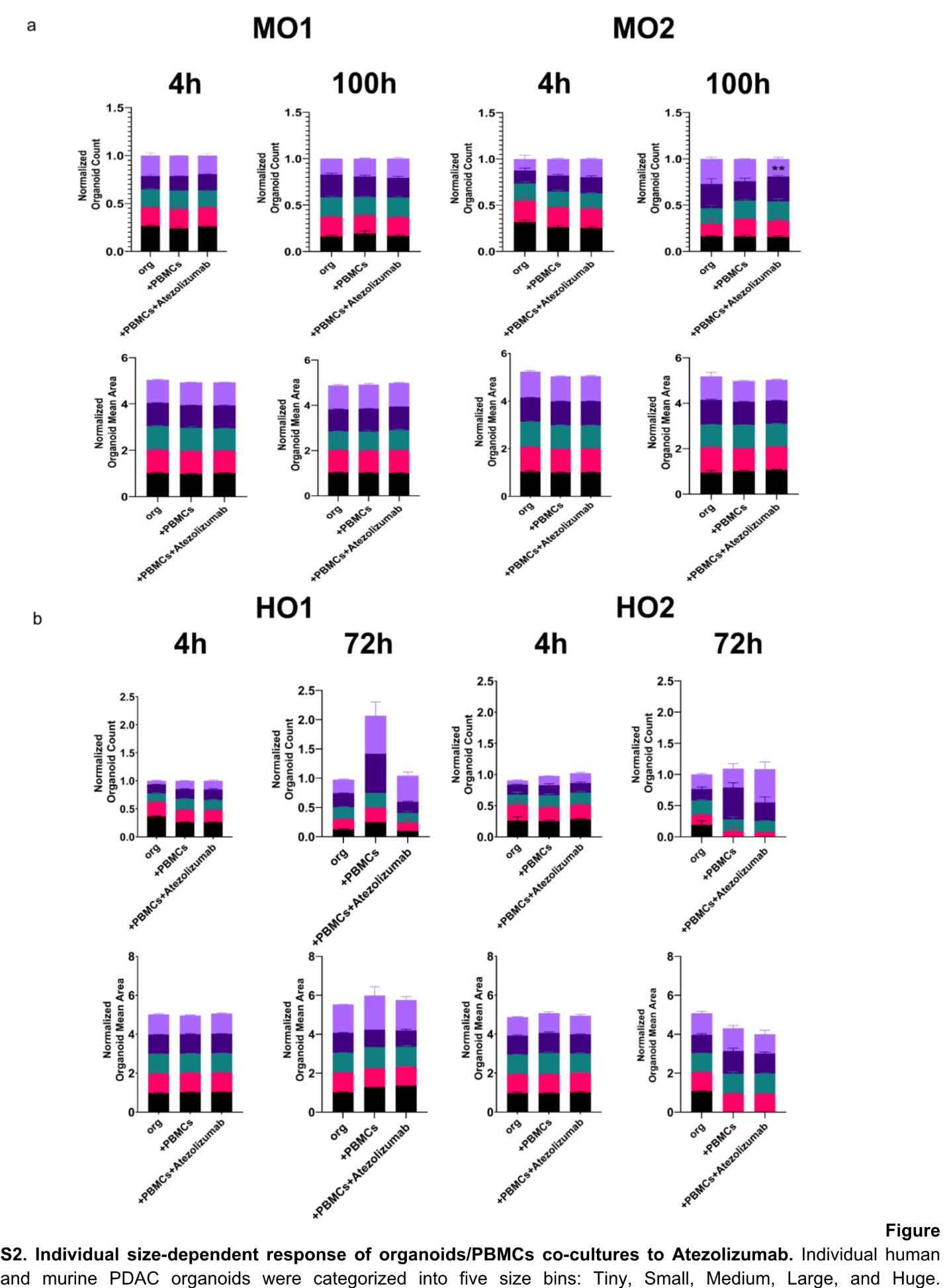


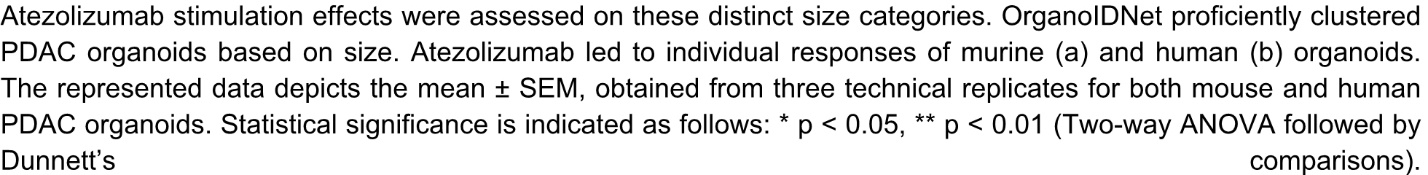


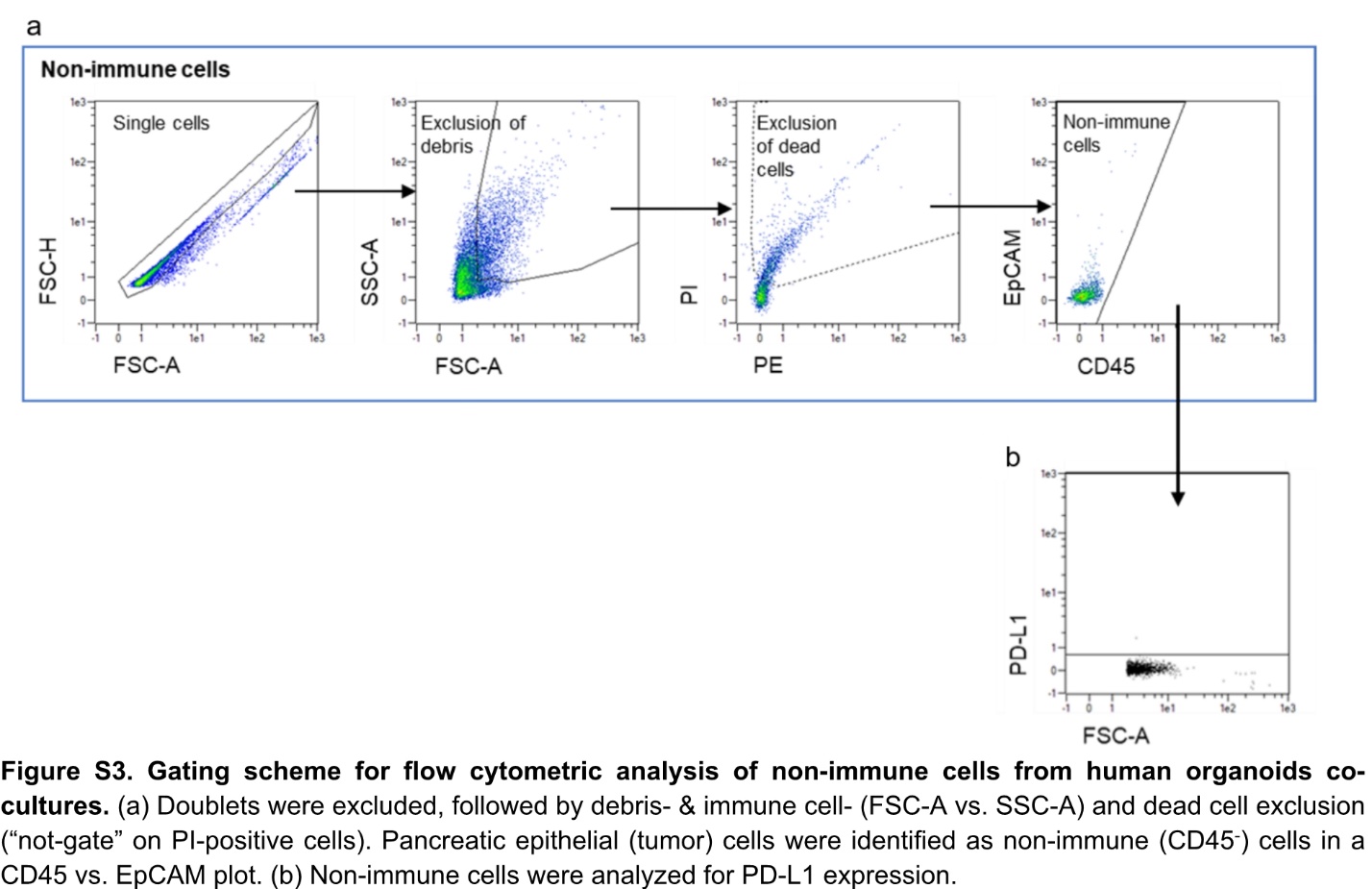


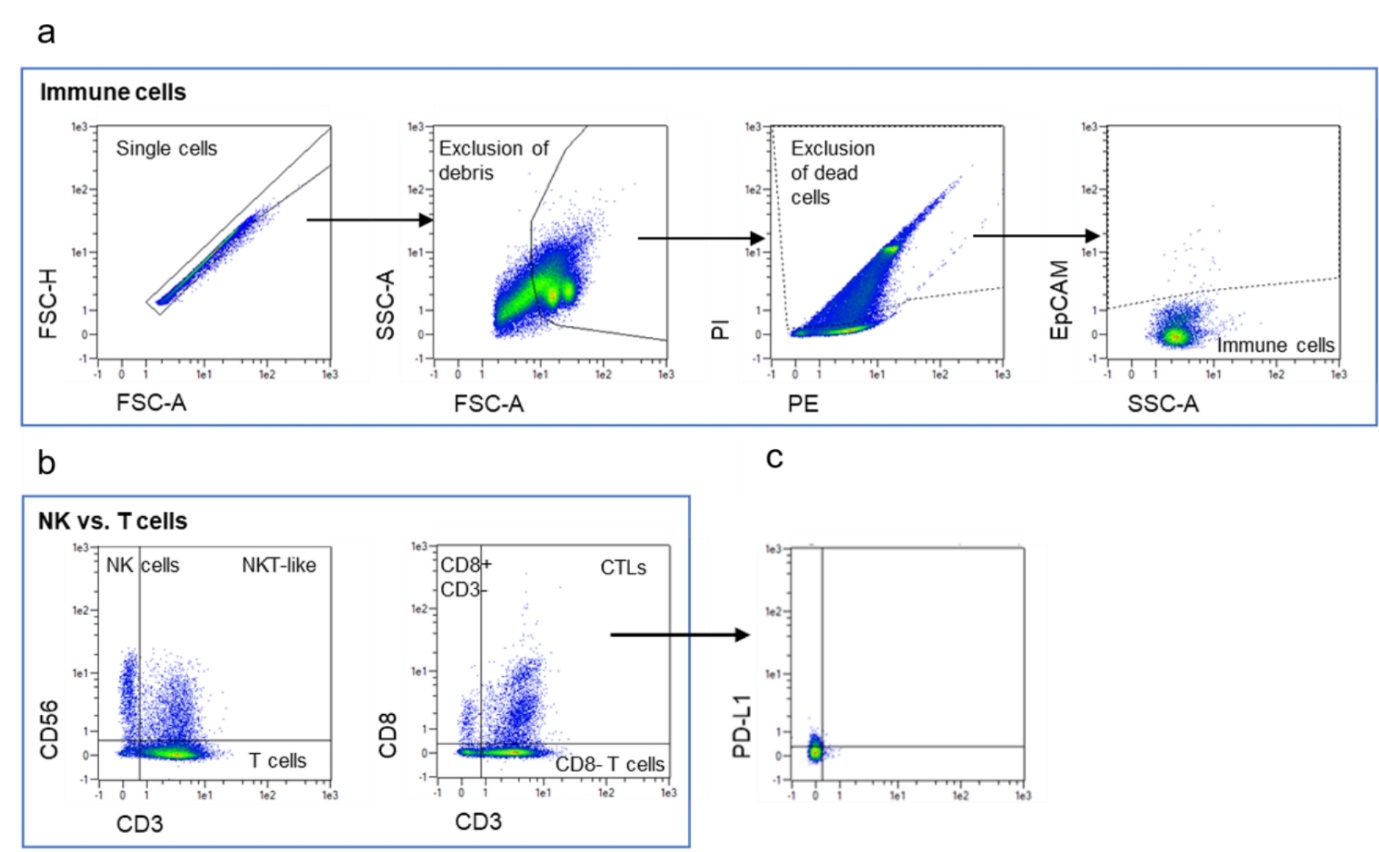


**Figure S4. Gating scheme for flow cytometric analysis of immune cell infiltration into human organoids in co-cultures.** (a) Doublets were excluded as the initial step via FSC-A vs. FSC-H, followed by debris (based on low size and granularity; FSC-A vs. SSC-A) and exclusion of dead cells (dashed “not-gate” on PI-positive cells). Immune cells were identified as EpCAM^−^ cells (“not-gate” in SSC-A vs. EpCAM). (b) Among the EpCAM^−^ cells, CD3 vs. CD56 expression was used to discriminate between T cells and NK cells. Moreover, cytotoxic T cells were identified in a CD3 vs. CD8 plot again based on viable EpCAM^−^ cells. (c): PD-L1 was assessed from CTLs positive cells.
